# ACAT1 regulates tertiary lymphoid structures and correlates with immunotherapy response in non–small cell lung cancer

**DOI:** 10.1172/JCI181517

**Published:** 2025-04-01

**Authors:** Mengxia Jiao, Yifan Guo, Hongyu Zhang, Haoyu Wen, Peng Chen, Zhiqiang Wang, Baichao Yu, Kameina Zhuma, Yuchen Zhang, Jingbo Qie, Yun Xing, Pengyuan Zhao, Zihe Pan, Luman Wang, Dan Zhang, Fei Li, Yijiu Ren, Chang Chen, Yiwei Chu, Jie Gu, Ronghua Liu

**Affiliations:** 1 Shanghai Fifth People’s Hospital, and Shanghai Key Laboratory of Medical Epigenetics, Institutes of Biomedical Sciences, Fudan University, Shanghai, China.; 2 Department of Thoracic Surgery, Zhongshan Hospital, Fudan University, Shanghai, China.; 3 Department of Thoracic Surgery, Changzheng Hospital, Naval Medical University, Shanghai, China.; 4 Department of Neurology, Children’s Hospital of Fudan University, Shanghai, China.; 5 Department of Immunology, School of Basic Medical Sciences, and MOE Innovative Center for New Drug Development of Immune Inflammatory Diseases, Fudan University, Shanghai, China.; 6 Department of Pathology and Frontier Innovation Center, School of Basic Medical Sciences, Fudan University, Shanghai, China.; 7 Department of Thoracic Surgery, Shanghai Pulmonary Hospital, Tongji University School of Medicine, Shanghai, China.

**Keywords:** Immunology, Oncology, Cancer immunotherapy, Carbohydrate metabolism, Cell stress

## Abstract

Tertiary lymphoid structures (TLS) in the tumor microenvironment (TME) are emerging solid-tumor indicators of prognosis and response to immunotherapy. Considering that tumorigenesis requires metabolic reprogramming and subsequent TME remodeling, the discovery of TLS metabolic regulators is expected to produce immunotherapeutic targets. To identify such metabolic regulators, we constructed a metabolism-focused sgRNA library and performed an in vivo CRISPR screening in an orthotopic lung tumor mouse model. Combined with The Cancer Genome Atlas database analysis of TLS-related metabolic hub genes, we found that the loss of *Acat1* in tumor cells sensitized tumors to anti-PD1 treatment, accompanied by increased TLS in the TME. Mechanistic studies revealed that ACAT1 resulted in mitochondrial protein hypersuccinylation in lung tumor cells and subsequently enhanced mitochondrial oxidative metabolism, which impeded TLS formation. Elimination of ROS by NAC or *Acat1* knockdown promoted B cell aggregation and TLS construction. Consistently, data from tissue microassays of 305 patients with lung cancer showed that TLS were more abundant in non–small cell lung cancer (NSCLC) tissues with lower *ACAT1* expression. Intratumoral *ACAT1* expression was associated with poor immunotherapy outcomes in patients with NSCLC. In conclusion, our results identified ACAT1 as a metabolic regulator of TLS and a promising immunotherapeutic target in NSCLC.

## Abstract 2

Loss of *Acat1* in NSCLC cells impedes TLS and anti-tumor immunotherapy ACAT1 increases intratumoral oxidative stress by promoting mitochondrial hypersuccinylation Reduction of oxidative stress promotes B cell aggregation to build the TLS ACAT1 correlates with TLS abundance and immunotherapeutic response of NSCLC patients

## Graphical abstract

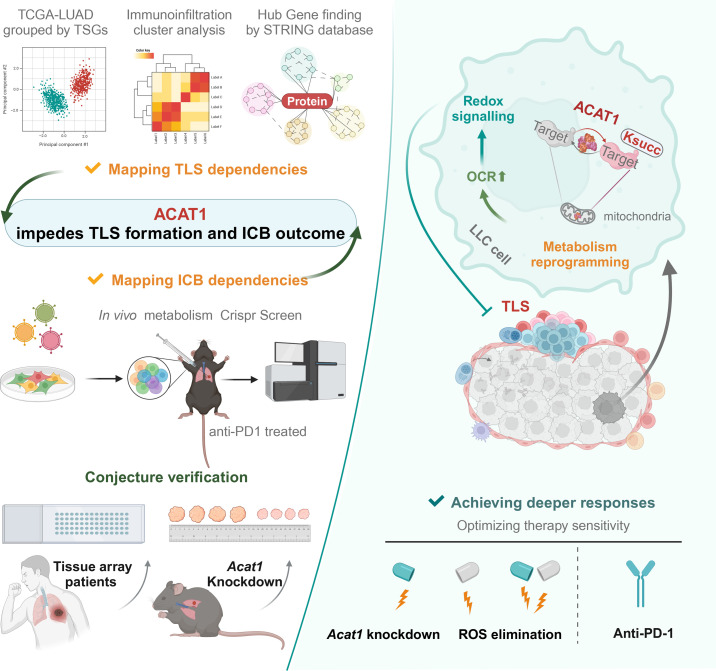

## Introduction

Tertiary lymphoid structures (TLS) in the tumor microenvironment (TME) phenotypically resemble conventional secondary lymphoid organs and play a crucial role in facilitating the influx of immune cells into the tumor site, marking the transition from an immunologically “cold” to a “hot” TME. The presence of TLS is associated with prolonged patient survival and favorable responses to current cancer therapies, including immune checkpoint inhibitors (ICI) (1, 2). Within TLS, the interaction between B cells and T follicular helper cells is particularly intense, creating a unique niche that supports the clonal diversity, selective expansion, and differentiation of B cells into IgG-producing plasma cells (3). This leads to an increase in tumor antigen–specific antibodies and enhances the efficacy of ICI in cancers such as renal cell carcinoma (RCC) and non–small cell lung cancer (NSCLC) (4, 5). Despite their importance, the drivers of TLS formation and their contribution to intratumoral immune responses remain incompletely characterized.

The TME is a complex ecosystem where tumor cells and immune cells coexist and share a common metabolic milieu. Metabolic reprogramming in tumor cells not only meets the increased energy demands for rapid proliferation, invasion, and metastasis but also profoundly influences immune cell function within the same niche (6). Tumor metabolism can suppress immune responses by releasing immunosuppressive metabolites such as lactate, PGE2, arginine, and ROS, which cause metabolic stress in the TME (7–9). Additionally, tumor cells compete with immune cells for essential nutrients, further impairing antitumor immunity. While the role of tumor metabolism in immune suppression is well documented, its impact on TLS formation and related immune responses remains underexplored.

Here, we used CRISPR screening of metabolism-associated genes and found that mitochondrial acetyl-CoA acetyltransferase (ACAT1) connects tumor cell metabolism with TLS abundance. ACAT1 accelerated oxidative metabolism and ROS release in NSCLC cells by promoting succinylation of multiple mitochondrial proteins. Increased oxidative stress in the TME impeded B cell aggregation and TLS formation. Additionally, in patients with NSCLC, *ACAT1* expression was negatively correlated with TLS abundance. *Acat1* knockdown improved B cell and TLS abundance and increased the sensitivity of NSCLC cells to anti-PD1 immunotherapy, providing metabolic-intervention ideas for improving NSCLC immunotherapy.

## Results

### In vivo CRISPR screening for metabolic regulators of TLS in NSCLC identifies ACAT1.

**Figure 1:**
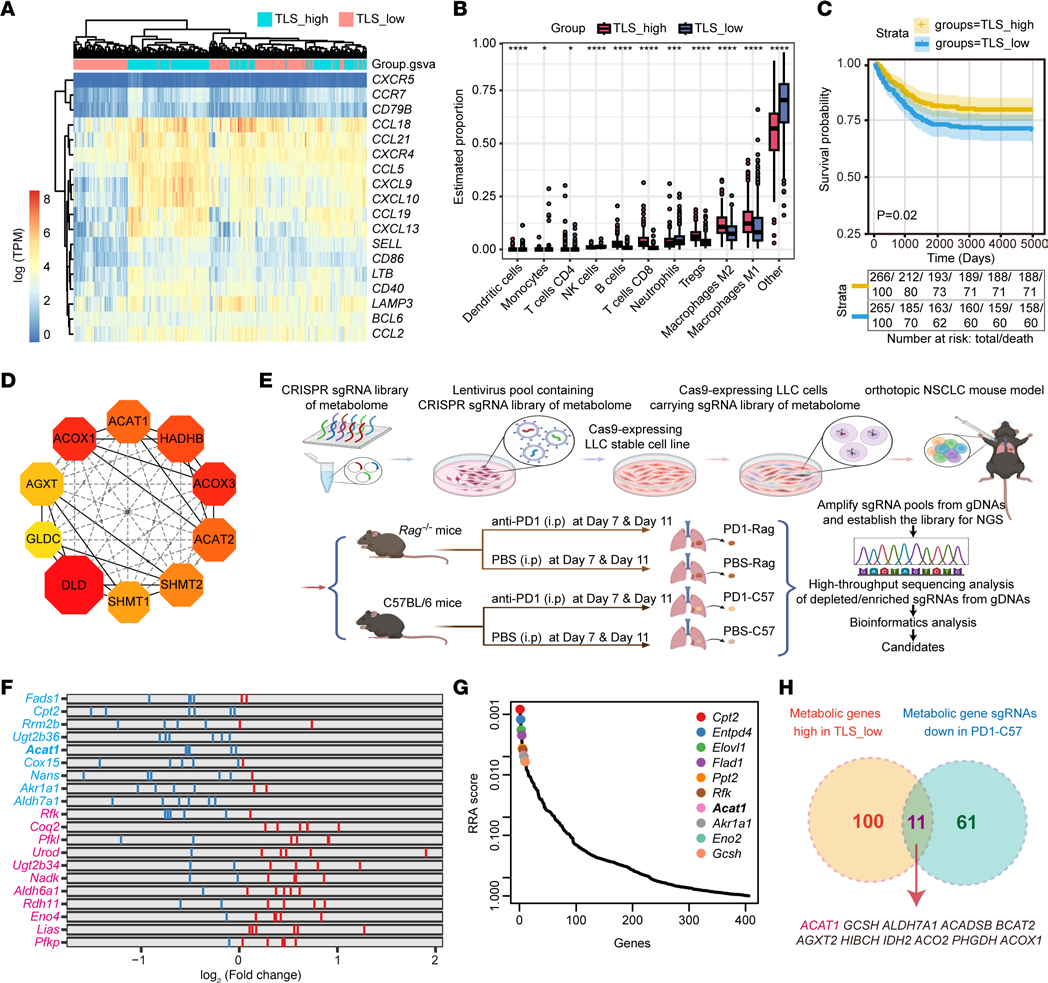
In vivo CRISPR screening for metabolic regulators of tertiary lymphoid structures in non–small cell lung cancer. (**A**) Expression of 18 tertiary lymphoid structure (TLS) signature genes (TSGs) in 533 patients from the The Cancer Genome Atlas_lung adenocarcinoma (TCGA_LUAD) cohort. Each column represents an individual patient. Patients were divided into TLS_high and TLS_low subgroups based on the expression level of all TSGs. (**B**) TME cell infiltrating characteristics of TLS_high group and TLS_low group (analyzed by quanTIseq). (**C**) Survival analyses for TLS_high group and TLS_low group in the TCGA_LUAD cohort using Kaplan–Meier curves. (**D**) Identification of hub genes from the protein–protein interaction network using the Maximal Clique Centrality algorithm. The edges represent protein–protein associations. (**E**) Strategy of in vivo metabolism-wide CRISPR screening. (PD1-Rag/PBS-Rag, *n* = 5; PD1-C57/PBS-C57, *n* = 12). (**F**) Comparison of PBS-C57 and PD1-C57 genes whose knockout (KO) can enhance (blue) or inhibit (red) sensitivity to anti-PD1 treatment (Top 10). Each gene with 6 sgRNAs. (**G**) Schematic representation of the top 10 candidates whose KO is expected to enhance or inhibit anti-PD1 treatment from **B**. RRA, Robust Rank Aggregation. (**H**) Venn diagram showing the metabolic genes as immunotherapy candidates.

To investigate tumor characteristics affecting TLS, we analyzed gene expression profile of lung adenocarcinoma (LUAD) in The Cancer Genome Atlas (TCGA) dataset (Supplemental Table 1; supplemental material available online with this article; https://doi.org/10.1172/JCI181517DS1). We constructed a signature from a compendium of TLS signature genes (TSGs, *CXCR5, CCR7, CD79B, CCL18, CCL21, CXCR4, CCL5, CXCL9, CXCL10, CCL19, CXCL13, SELL, CD86, LTB, CD40, LAMP3, BCL6, CCL2*) (10–12), and the 533 patients in the metacohort were divided into 2 subgroups using R package “GSVA” based on the expression level of all TSGs (Figure 1A and Supplemental Figure 1A). Patients with high expression of TSGs displayed a high level of immune cell infiltration, including B cells, CD8^+^ T cells, and M1 macrophages (Figure 1B). A high TLS abundance was associated with prolonged patient survival (Figure 1C). As expected, the highly expressed genes within the TLS-high group were related to immunity pathways, such as the B cell receptor signaling pathway, while highly expressed genes within the TLS-low groups were mainly related to the metabolic pathways, such as carbon metabolism, TCA, and fatty acid metabolism (Supplemental Figure 1, B and C). We identified 10 top hub genes related to metabolism using the Search Tool for the Retrieval of Interacting Genes/Proteins (STRING) Database (13) and Maximal Clique Centrality (MCC) arithmetic (Figure 1D and Supplemental Figure 1D).

Given the role of TLS in tumor immunotherapy responsiveness, to search for metabolic regulators for TLS function, we developed an in vivo CRISPR screening using an orthotopic NSCLC mouse model with anti-PD1 immunotherapy. First, we constructed a metabolism-focused sgRNA library containing 406 metabolic regulators and 43 nontargeting guides (Supplemental Table 2). After confirming that the guides were evenly distributed (Supplemental Figure 1, E and F), we generated Lewis lung carcinoma (LLC) cell lines with or without Cas9 expression. These cells were infected with a lentivirus to express the sgRNAs and injected into the mouse lung to establish an orthotopic lung tumor model. On days 7 and 11, the mice were treated with anti-PD1 or isotype control, and the tumors were harvested on day 15. Genomic DNA was isolated, and amplified sgRNA samples were prepared for next-generation sequencing (NGS) (Figure 1E). The top 10 genes whose knockout could enhance (blue) or inhibit (red) the sensitivity to anti-PD1 treatment are displayed (Figure 1F and Supplemental Figure 1G).

The results showed that 61 genes bore significantly different sgRNA abundance in mice treated with anti-PD1 antibody compared with the control group, indicating that they were not suitable for immunotherapy (Supplemental Table 3, Figure 1G, and Supplemental Figure 1H). Among these genes, 11 overlapped with the highly expressed metabolic genes in patients within TLS-low group (Figure 1H). Interestingly, *Acat1* ranked highly both in Robust Rank Aggregation score for anti-PD1 treatment-depleted sgRNA and in metabolic hub genes correlated with TLS (Figure 1, D and G).

Tetrameric ACAT1 is commonly upregulated in cells stimulated by EGF, and, in diverse human cancer cells, inhibition of tetrameric ACAT1 attenuated tumor growth (14). However, ACAT1 inhibits glioma tumorigenesis through catalyzing glycine decarboxylase K514 acetylation (15), which suggests the role of ACAT1 in tumor progression is still a matter of debate and warrants further investigation (15).

### Patients with high ACAT1 levels have fewer TLS and poorer responses to immunotherapy.

**Figure 2:**
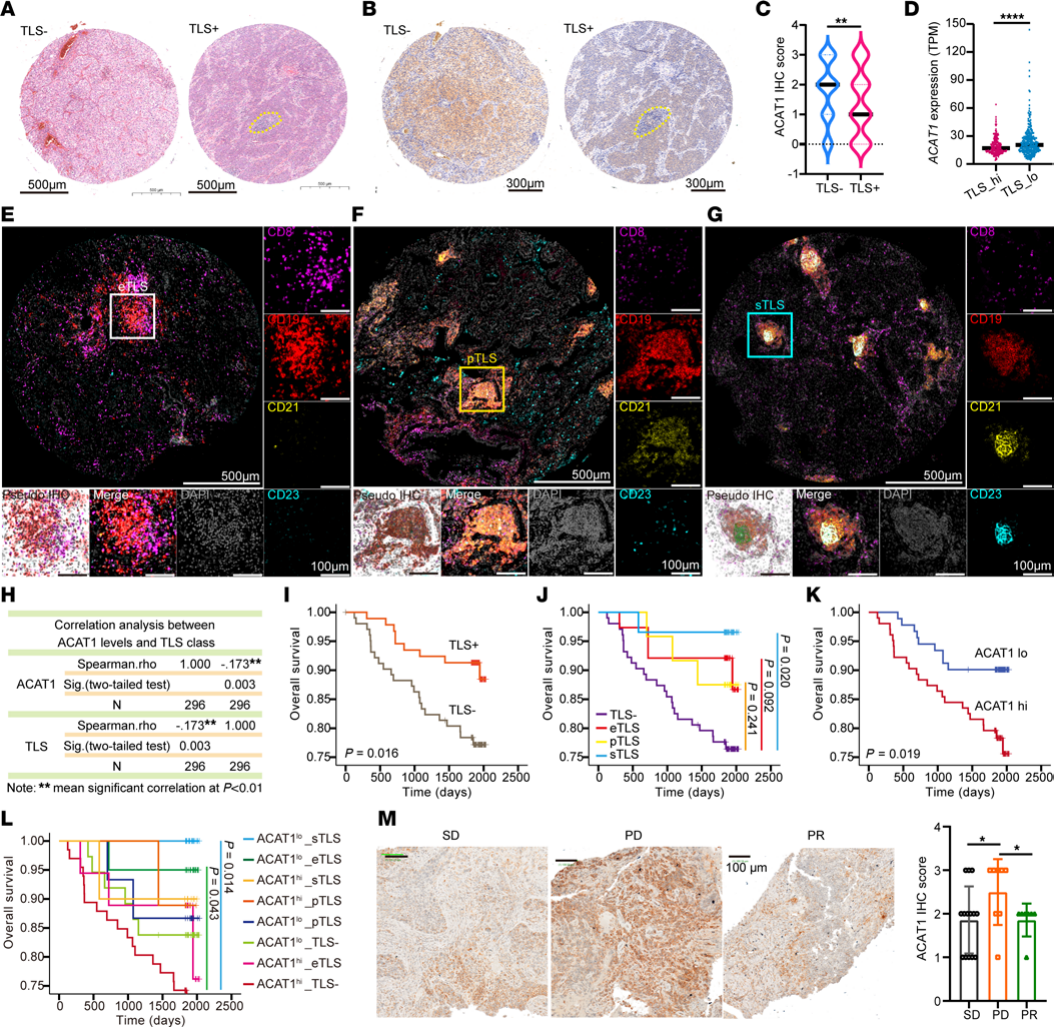
ACAT1 correlates with tertiary lymphoid structure abundance and non–small cell lung cancer immunotherapeutic response in patients. (**A**) Tertiary lymphoid structures (TLS) in 305 human lung cancer specimens were observed using H&E. (**B** and **C**) IHC analyses of ACAT1 levels of 305 human lung cancer specimens were performed. (**D**) Expression of *ACAT1* in the TLS_high group and TLS_low group in Figure 1A. (**E**–**G**) TLS maturity in 305 human lung cancer specimens were observed using immunofluorescence staining. Purple, CD8; red, CD19; yellow, CD21; blue, CD23; and gray, DAPI for nucleus. (**E**) Immature early TLS (eTLS). (**F**) Primary follicle-like TLS (pTLS). (**G**) Secondary follicle-like TLS (sTLS). (**H**) Correlation analysis between ACAT1 levels and TLS class in 305 human lung cancer specimens. (**I**–**L**) Kaplan-Meier plots of the overall survival durations of the patients (*n* = 194). (**I**) The patients were divided into 2 groups with TLS+ (*n* = 91) and TLS– (*n* = 103). (**J**) The patients were divided into 4 groups with eTLS (*n* = 38)/pTLS (*n* = 24)/sTLS (*n* = 29)/TLS– (*n* = 103). (**K**). The patients were divided into 2 groups with ACAT1 low expression (*n* = 91) and ACAT1 high expression (*n* = 103) (**L**). The patients were divided into 8 groups with ACAT1^lo^ TLS– (*n* = 37); ACAT1^lo^ eTLS (*n* = 20); ACAT1^lo^ pTLS (*n* = 15); ACAT1^lo^ sTLS (*n* = 19); ACAT1^hi^ TLS– (*n* = 66); ACAT1^hi^ eTLS (*n* = 18); ACAT1^hi^ pTLS (*n* = 9); ACAT1^hi^ sTLS (*n* = 10). (**M**) IHC analyses of ACAT1 expression of 29 human lung cancer specimens that received immunotherapy were performed. Representative images are shown. Data are shown as the mean ± SD. **P < 0.05; **P < 0.01; ****P < 0.0001 with Mann-Whitney test (**C**, **D**, and **M**) and log-rank test (**I**–**L**).

To confirm that ACAT1 was correlated with TLS, we used an existing array constructed from 305 tissue samples of patients with lung cancer to determine the TLS counts and *ACAT1* expression (Supplemental Table 4) (16). The presence of TLS was initially assessed based on the clinicopathological characteristics analyzed by H&E staining (Figure 2A). ACAT1 antibody-based IHC was performed to analyze the relationship between ACAT1 levels and TLS in patients with NSCLC (Figure 2B). Patients with TLS exhibited a decreased level of ACAT1 within tumors compared with those without TLS (Figure 2C). TCGA data analysis also showed that *ACAT1* expression levels were higher in patients with LUAD bearing lower TLS (Figure 2D). TLS maturation was further evaluated using a 5-color multispectral immunofluorescence (mIF) technique (CD8/CD19/CD21/CD23/DAPI) (17) (Figure 2, E–G). The selection of mIF markers was based on the fact that the maturation process of TLS starts with immature early TLS (eTLS), progresses to primary follicle-like TLS (pTLS) characterized by the emergence of a CD21-positive follicular dendritic cell (FDC) network, and further evolves into secondary follicle-like TLS (sTLS) stage with CD23-expressing germinal center (10, 18, 19). Introducing the maturity status of TLS into analysis, patients were classified into 4 distinct groups (TLS–, eTLS, pTLS, and sTLS). Results showed that the ACAT1 level was negatively correlated with the TLS class in patients with NSCLC (Figure 2H).

A total of 111 patients were excluded from the analysis examining the prognostic influence of TLS biology in NSCLC due to either limited access to prognostic information or having undergone nonsurgical treatment during the follow-up period. The results revealed a statistically significant improvement in prognosis for patients harboring TLS (TLS+) compared with those without TLS (TLS–) (Figure 2I). In addition, we identified that patients with mature sTLS exhibited the best prognosis. Both pTLS and eTLS showed trends toward better prognosis compared with TLS–, although the difference was not significant when compared with the sTLS (Figure 2J). These results highlight that TLS displays a good prognosis in which sTLS contributes most prominently to this positive prognostic outcome. Subsequently, the joint prognostic analysis of TLS and ACAT1 expression was performed. Results showed that lower ACAT1 levels implied better positive clinical outcomes (Figure 2K). Patients characterized by sTLS and low ACAT1 expression had the most favorable prognosis, whereas those with TLS– and high ACAT1 expression showed the poorest prognosis (Figure 2L).

Tumor puncture samples from 29 patients with NSCLC treated with immunotherapy were collected, and ACAT1 antibody-based IHC revealed that patients with a poor immunotherapy response (PD, disease progression) had significantly higher ACAT1 levels than those who responded well to immunotherapy (SD or PR, disease stability or remission) (Figure 2M and Supplemental Table 4). These data indicated that ACAT1 is involved in the regulation TLS-related tumor immunity.

### Acat1 knockdown improves the TLS abundance and sensitivity to immunotherapy.

**Figure 3:**
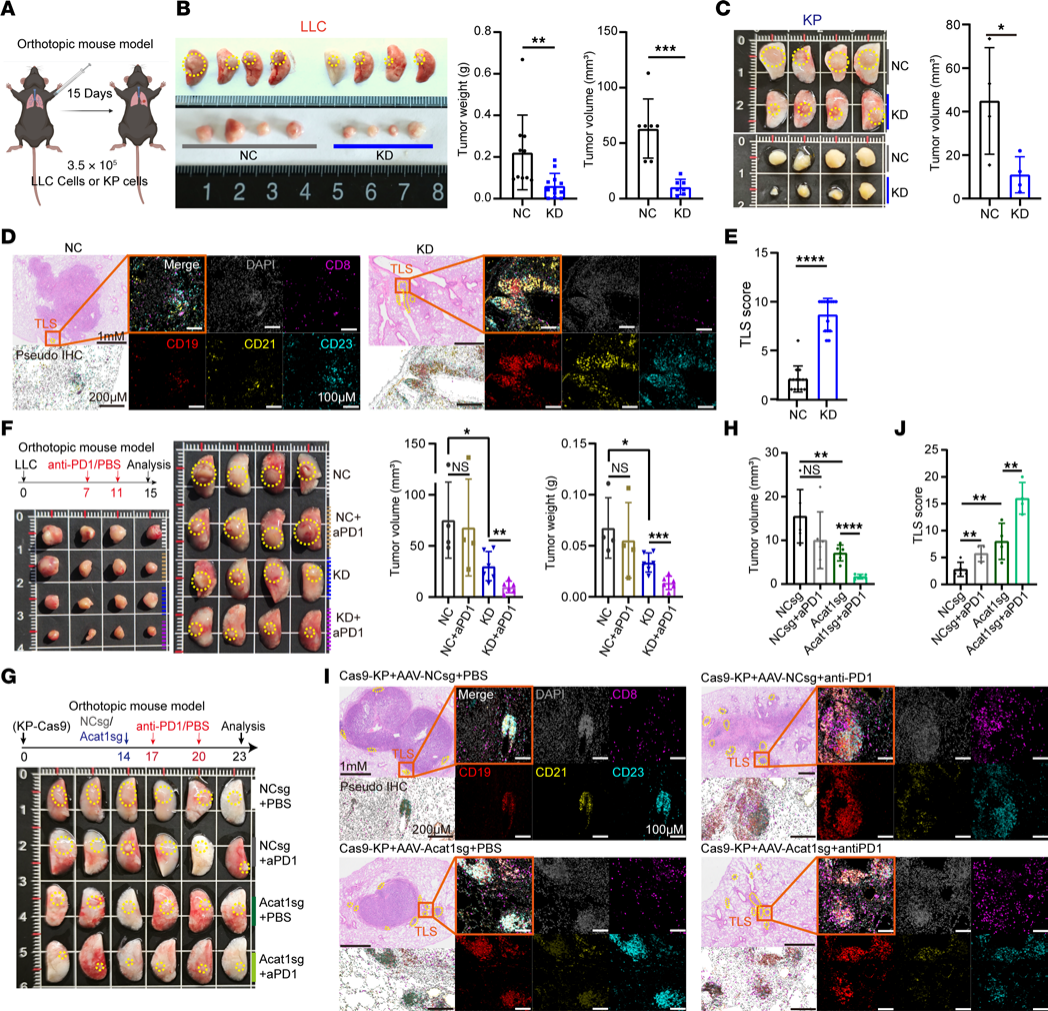
*Acat1* knockdown in lung carcinoma cells improves tertiary lymphoid structure abundance and sensitivity to immunotherapy. (**A**–**C**) Tumor development was measured in orthotopic lung tumor mice models. Schematic diagram (**A**). *Acat1* negative control (NC) and knockdown (KD) lung carcinoma cells were inoculated on the lungs of C57/BL6. LLC model, NC and KD *n* = 10 and 12, respectively, experimental replicates = 3. (**B**). KP model, *n* = 4 per group, experimental replicates = 2. (**C**). (**D** and **E**) Tertiary lymphoid structures (TLS) in mice lung tissues of KP model were observed using H&E and immunofluorescence. Purple, CD8; red, CD19; yellow, CD21; blue, CD23; and gray, DAPI for nucleus. Representative staining images are shown (**D**). TLS score of lung tissues in NC and KD groups were determined by the number and maturity of TLS as well as the ratio of the total area of all TLS to the total tumor area, *n* = 14 per group (**E**). (**F**) Tumor development for NC and KD groups with or without anti-PD1 treatment was measured in LLC model, NC and KD *n* = 4 and 6, respectively, experimental replicates = 2. (**G** and **H**) Tumor development for NC sg and Acat1 sg groups with or without anti-PD1 treatment was measured in KP-cas9 model, *n* = 6 per group. (**I** and **J**) TLS in **G** were observed using H&E and immunofluorescence. Purple: CD8; red: CD19; yellow: CD21; blue: CD23; and grey: DAPI. Representative staining images are shown, *n* = 4 per group. (**I**). TLS score was determined by the number and maturity of TLS as well as the ratio of the total area of all TLS to the total tumor area (**J**). Data are shown as the mean ± SD. *P < 0.05; **P < 0.01; ***P < 0.001; ****P < 0.0001 with 2-tailed unpaired Student’s *t* tests. Please also see Supplemental Figure 2.

Next, we used the *Kras*^G12D^/*Trp53*^−/−^ (KP) and LLC orthotopic lung tumor model to investigated the regulatory role of ACAT1 in TLS formation and related antitumor immunity, as described previously (20–23). We generated LLC and clonal KP mouse lung cancer cell lines with or without *Acat1* knockdown (KD or negative control [NC]) and confirmed ACAT1 deficiency (Supplemental Figure 2, A and B). NC or KD LLC/KP cells were injected into mouse lungs to establish an orthotopic lung tumor model (24, 25). The results showed that specific *Acat1* KD in LLC cells suppressed tumor development, with tumor volume and weight being significantly lower in the KD than in the control NC group; the same conclusion was validated in the KP model (Figure 3, A–C). Next, *Acat1^–/–^* mice were generated (Supplemental Table 5), and their lungs, alongside lungs of WT (*Acat1^+/+^*) mice, were injected with NC LLC cells to construct orthotopic lung tumor models. Tumorigenesis was significantly faster in *Acat1^–/–^* mice compared with their *Acat1^+/+^* counterparts (Supplemental Figure 2, C and D), whereas, in *Rag1^–/–^* mice without T and B lymphocytes, tumor development did not differ significantly between the NC and KD LLC groups (Supplemental Figure 2, E and F). These results indicate that ACAT1 affects tumor development in an immune system–dependent manner.

To investigate whether TLS were involved in tumor suppression caused by *Acat1* KD, H&E and CD19/CD8 IF staining of tumor and adjacent normal tissues were performed to identified TLS (Supplemental Figure 2, G and H). Furthermore, the 5-color mIF was employed to investigate whether ACAT1 impacts the TLS score, which encompasses TLS maturity (Figure 3, D and E). As expected, TLS numbers were increased in KD tumor lung cancer tissues compared with those in the control NC group, while TLS infiltration in *Acat1*^–/–^ mice was not obvious (Supplemental Figure 2, D and I). Consistently, gene cluster analysis showed that KD and NC LLC cells displayed different expression levels of immune-related genes and biological processes, such as chemotaxis, proliferation, and activation of leukocytes as well as cytokine-mediated signaling pathways (Supplemental Figure 2, J–L).

Since TLS is related to enhanced tumor immunotherapy, we next confirmed the role of ACAT1 in antitumor immunotherapy. NC and KD LLC cells were injected into mouse lungs to establish orthotopic lung tumor models, followed by anti-PD1 therapy on days 7 and 11. Tumor development was detected after 15 days. The results showed that ACAT1 knockdown repressed tumor development. There were no significant changes in tumor weight or size in the NC mice treated with or without anti-PD1 therapy. Tumors were dramatically reduced in the KD mice treated with anti-PD1 (Figure 3F). These results indicated that *Acat1* KD increased the sensitivity of LLC tumors to anti-PD1 immunotherapy. Additionally, combination therapy with *Acat1* KD and anti-PD1 treatment maximally enhanced the TLS scores in lung tumors (Supplemental Figure 2M). In order to validate the therapeutic benefits of inducing ACAT1 deletion in combination with immunotherapy during tumor progression, we utilized the previously described KP-CAS9 cell line to establish a KP-CAS9 mouse model (20, 23). Fourteen days after tumor induction, adeno-associated viruses (AAV) viruses carrying *Acat1* sgRNA/NC sgRNA were injected peritumorally to specifically knock out *Acat1* in tumors. This was followed by anti-PD1 therapy at days 17 and 20, and tumor development was detected at day 23 (Figure 3, G–J). Similarly, the synergistic approach of *Acat1*-induced knockout coupled with anti-PD1 therapy exhibited maximal efficacy in suppressing tumor progression and substantially elevated TLS score.

### B cell infiltration and activation are promoted within lung tumors upon Acat1 KD.

**Figure 4:**
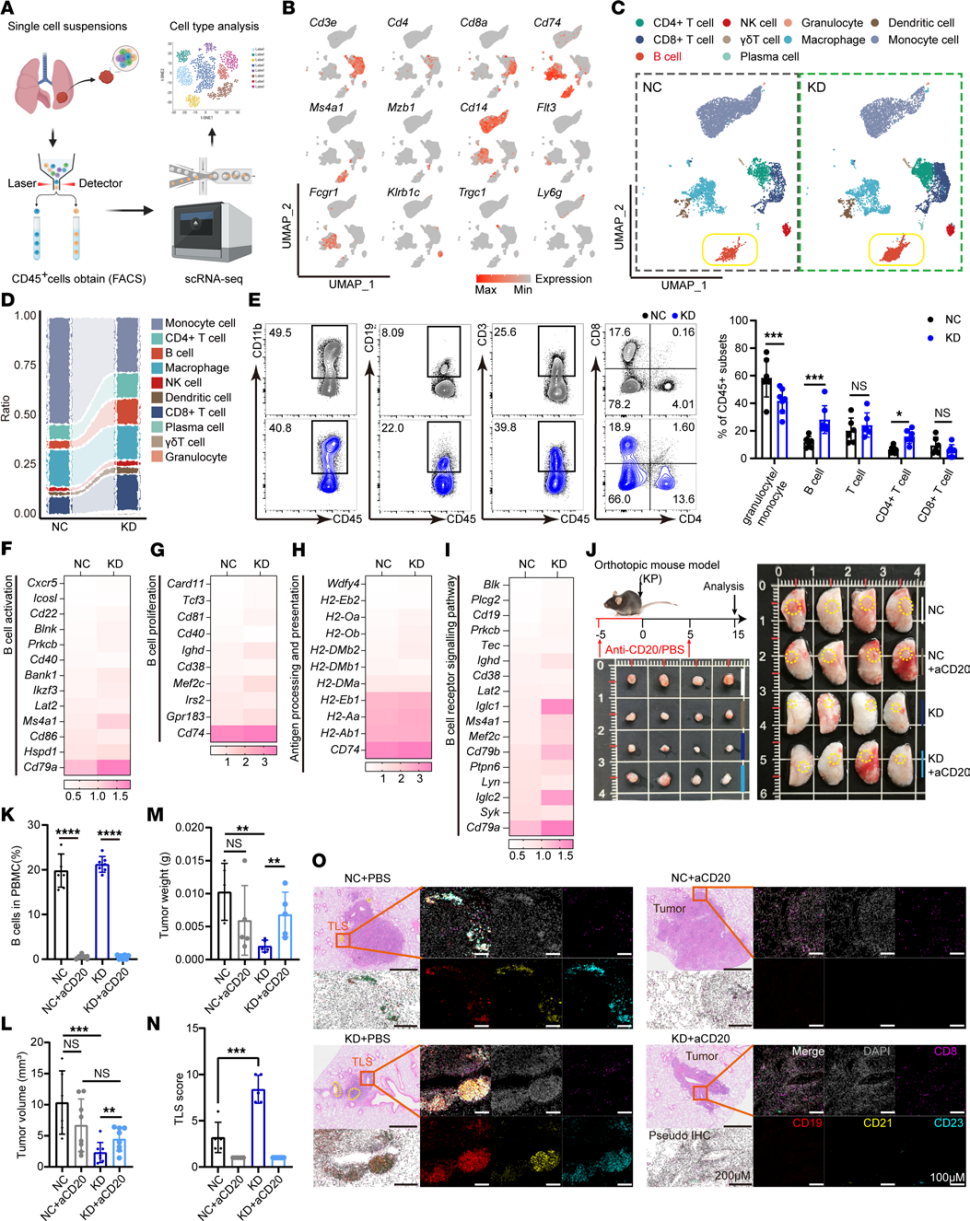
*Acat1* knockdown promotes the infiltration and activation of B cells in the tumor microenvironment. (**A**–**D**) CD45^+^ immune cells were sorted by flow cytometry from tumor isolated from orthotopic lung tumor mice models. Single-cell sequencing was performed (**A**), canonical markers of different cell types were used to identify cell clusters (**B**), and cell clusters are displayed (**C**). The proportion of different immune cell subsets in the 2 groups (pool of 3 mice per group) (**D**). (**E**) The proportion of different immune cell subsets in mouse tumor were verified by flow cytometry, *n* = 7 per group, experimental replicates = 2. (**F**–**I**) Gene-expression levels related to B cell activation (**F**), proliferation (**G**), antigen processing and presentation (**H**), and B-cell receptor signaling pathway of *Acat1* negative control (NC) and knockdown (KD) groups. (**J**–**M**) Anti-CD20 antibodies were used to eliminate B cells in the KP model, *n* = 4 per group, experimental replicates = 2. (**J**), followed by determination of the B cell depletion effect (**K**), tumor volume (**L**), tumor weight (**M**). (**N** and **O**) Tertiary lymphoid structures (TLS) in mouse lung tissues of **J** were observed using H&E and immunofluorescence. Purple, CD8; red, CD19; yellow, CD21; blue, CD23; and grey, DAPI for nucleus. Representative staining images are shown (**O**). TLS score was determined by the number and maturity of TLS as well as the ratio of the total area of all TLS to the total tumor area, *n* = 5 per group. (**N**). Data are shown as the mean ± SD. *P < 0.05; **P < 0.01; ***P < 0.001; ****P < 0.0001 with 2-tailed unpaired Student’s *t* tests. Please also see Supplemental Figure 3.

Given the heterogeneity of tumor-infiltrating immune cells, we performed single-cell RNA-seq (scRNA-seq) on lung tumor specimens to provide a comprehensive and unbiased assessment of the immune responses affected by *Acat1* KD. We harvested tumors separately from mice that were burdened with NC and KD LLC cells, and single cell suspensions were collected and directly analyzed on a 10X Genomics platform (Figure 4A). We identified 17 distinct clusters using a standard procedure and further annotated them into 10 cell types: B Cells, CD4^+^ T cells, CD8^+^ T cells, dendritic cells, granulocytes, macrophages, monocytes, natural-killer cells, plasma cells, and γδT cells (Figure 4, B and C). Consistent with our previous observations that TLS increased, there was an increase in B cell clusters in *Acat1* KD tumors, combined with more CD4^+^ T follicular helper (TFH) cells (Figure 4D). We confirmed this increase in KD compared with NC tumors using flow cytometry (Figure 4E and Supplemental Figure 3, A and B).

B cells are considered to be the main TLS cell population (12, 26), and B cell–mediated formation of germinal centers is a marker of TLS maturation. B cells in germinal cancers participate in antitumor immunity through antigen presentation, which activates CD4^+^ TFH cells. Consistently, genes related to B cell activation, proliferation, and antigen-presentation, as well as B cell signaling pathways were upregulated in *Acat1* KD tumors (Figure 4, F–I). To further confirm the role of B cells in *Acat1* KD tumors, B cells were depleted using an anti-CD20 antibody before tumor implantation (Figure 4J and Supplemental Figure 3C). The depletion efficacy was subsequently verified by flow cytometry analysis of the B cell population in the PBMC of mice (Figure 4K and Supplemental Figure 3, D–F). *Acat1*-KD mice treated with anti-CD20 showed increases in tumor size compared with untreated mice (Figure 4, L and M and Supplemental Figure 3G). We observed that B cell elimination resulted in TLS disruption within the tissue (Figure 4, N and O and Supplemental Figure 3H). These results indicated that B cells were essential for TLS organization and responsive to tumor suppression introduced by *Acat1* KD. To investigate whether ACAT1 retained its antitumor efficacy in the restriction of TLS formation, we utilized TAK-799, an inhibitor of CCR5 and the CXCL13/CXCR5 axis (27, 28), all of which play a pivotal role in facilitating TLS development. Experimental results showed that administration of TAK-799 reduced the TLS score and increased tumor bearing in *Acat1*-KD mice (Supplemental Figure 3, I–K). These findings suggested that inhibiting the expression of ACAT1 within tumors enhanced the formation of TLS involving B cells, thereby inhibiting tumor progression.

### ACAT1 targets are involved in tumor mitochondrial metabolism and display hypersuccinylation.

**Figure 5:**
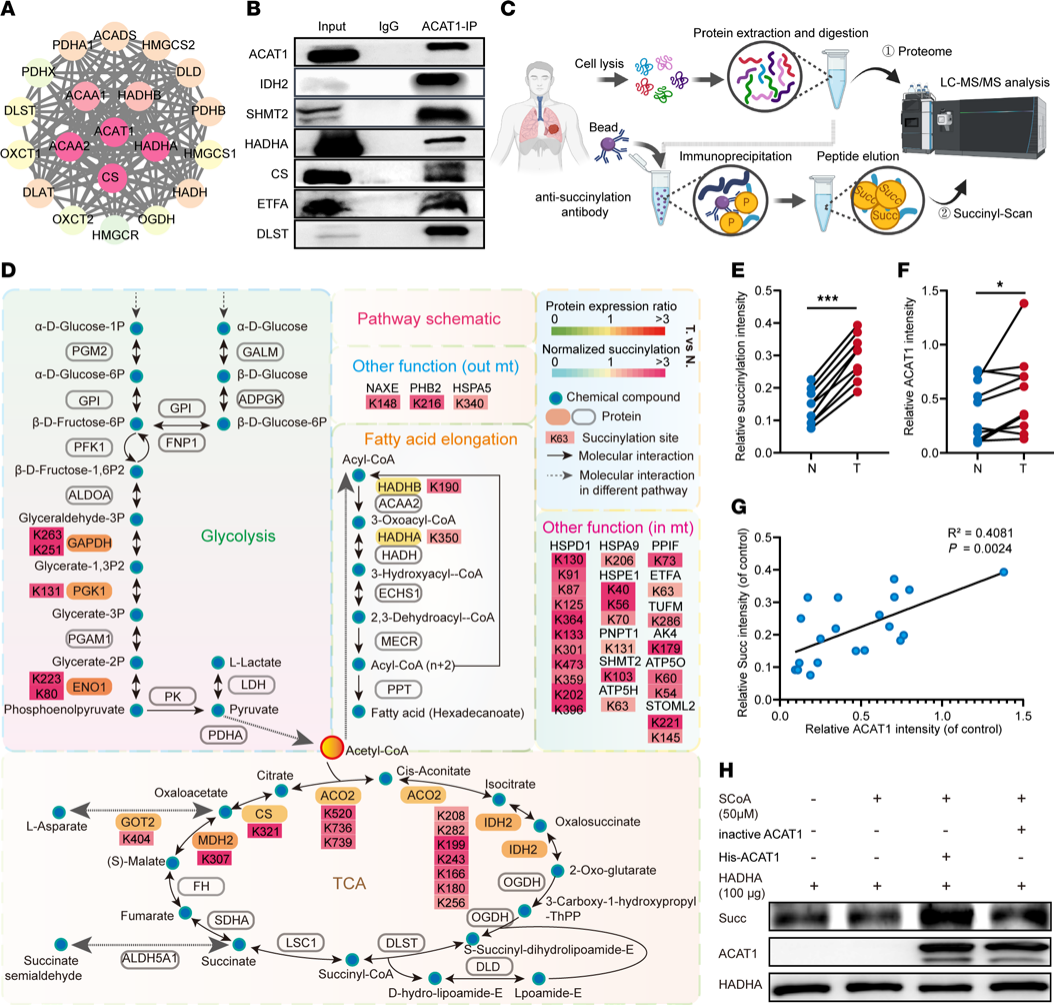
Mitochondrial ACAT1 targets display hypersuccinylation. (**A**) Protein interaction and enrichment analysis of ACAT1. The protein-protein interaction network of ACAT1 was constructed by the STRING/Proteins Database. (**B**) IP and IB analyses were performed with the indicated antibodies. (**C**) Workflow of the proteomic and succinylation modification proteomic detection. (**D**) Pathway schematic showing proteins with upregulated succinylation sites (Wilcoxon test, FDR *P* < 0.05) mapped onto key metabolic pathways. (**E**–**G**) Mitochondria were extracted from carcinoma (T) and adjacent tissue (N) from lung cancer patients, and mitochondria lysates of the mitochondria were prepared. The total mitochondrial-protein succinylation levels were analyzed by IB with the indicated antibodies. A comparison of mitochondrial-protein succinylation modification levels (**E**) and ACAT1 expression level (**F**) between carcinoma (T) and adjacent tissue (N) in Supplemental Figure 4C, *n* = 10 per group. Paired *t* tests were performed. A correlation analysis between the ACAT1 levels and of mitochondrial-protein succinylation levels in Supplemental Figure 4C (**G**). (**H**) ACAT1-mediated HADHA succinylation was analyzed by mixing purified ACAT1, HADHA, and succinyl-CoA. Heat-inactived ACAT1 was used as a negative control. The succinylation level of HADHA was detected by IB. *P < 0.05; ***P < 0.001. Please also see Supplemental Figure 4.

To identify potential ACAT1 targets in TLS regulation, we used the STRING database and ACAT1 immunoprecipitation coupled with mass spectrometry to identify ACAT1-interacting proteins (Figure 5, A and B and Supplemental Table 6). We found that 44 ACAT1 cofactors were related to metabolic pathways such as TCA and glycolysis (Supplemental Figure 4A and Supplemental Table 6). Interestingly, compared with our previous proteome data of 8 paired human lung cancers (29), we found that the identified ACAT1-binding proteins were hypersuccinylated at lysine residues in cancer tissues compared with normal lung tissues (Figure 5, C and D). Proteins with upregulated succinylation sites (USPs) in tumor tissues were mainly clustered in cell metabolic pathways involving the TCA and fatty acid metabolism (Figure 5D). Lysine succinylation (Ksucc) levels were differentially distributed across cellular compartments, and most USPs in lung cancer were also clustered inside mitochondria (Supplemental Figure 4B).

To assess the Ksucc levels of total mitochondrial proteins, we isolated mitochondria from normal and tumor tissues of 10 patients with lung cancer, and Ksucc levels were measured by Western blotting using a pan-anti-Ksucc antibody (Figure 5, E–G and Supplemental Figure 4, C–E). Consistent with the results of proteomic analysis, mitochondrial Ksucc levels were significantly upregulated in tumors compared with normal tissues (Figure 5E). ACAT1 levels were upregulated in tumors and exhibited a significant positive correlation with mitochondrial Ksucc level (Figure 5, F and G).

As some lysine acetyltransferases have succinyltransferase functions, we analyzed the affinity of ACAT1 for succinyl-CoA (SCoA). ACAT1 had a higher affinity score than CPT1A, a mitochondrial lysine succinyltransferase (mLSTase) (Supplemental Figure 4F) (30). Meanwhile, the expression of CPT1A and HAT1 (31), another mLSTase, showed no significant difference between tumor and paratumor mitochondria (Supplemental Figure 4, D and E). Affinity screening between mitochondrial proteins and SCoA could also be used for mLSTase research. Correlation analysis between the protein levels of the 8 potential mLSTases, and the mitochondrial-protein Ksucc sites revealed that ACAT1 levels were significantly positively associated with more Ksucc sites (Supplemental Figure 5A). AutoDock Vina and UCSF-chimera were used to analyze the interaction between ACAT1 and SCoA. The structures were analyzed by molecular replacement of the known structure of the ACAT1-CoA complex (Protein Data Bank ID: 2F2S) (Supplemental Figure 5B). Interestingly, we observed that several ACAT1 aa residues were responsible for the interaction of ACAT1 with SCoA, among which 385A was one of the most crucial sites, as it increased the nucleophilicity of active site Cys (32) and may facilitate succinyl group transfer (Supplemental Figure 5C).

To examine the effect of ACAT1 mitochondrial protein succinylation, we constructed stably *ACAT1* KD, overexpression (OE), and control 293T cell lines and derived in vitro succinylation assays using ACAT1 purified from OE cell lines (Supplemental Figure 5D). We isolated total mitochondrial proteins from *ACAT1* NC and KD cell lines and derived in vitro succinylation assays using ACAT1, purified from OE cell lines. Upregulation of Ksucc levels of mitochondrial proteins upon reaction with active ACAT1 compared with inactive ACAT1 was observed in both *ACAT1* NC and KD cell lines, whereas background Ksucc of mitochondrial proteins in KD cell lines was interestingly lower than what is seen in NC cell lines (Supplemental Figure 5E). To further investigate the effect of succinylation on proteins, we selected the ACAT1 target HADHA. In vitro succinylation assays showed that WT, but not heat-inactivated, ACAT1 succinylated HADHA in a dose-dependent manner (Figure 5H and Supplemental Figure 5F). These data indicated that ACAT1 functions as an mLSTase in mitochondria by promoting the succinylation of proteins involved in metabolic pathways.

### ACAT1 enhances tumor-cell oxidative metabolism in a succinylation-dependent manner.

**Figure 6:**
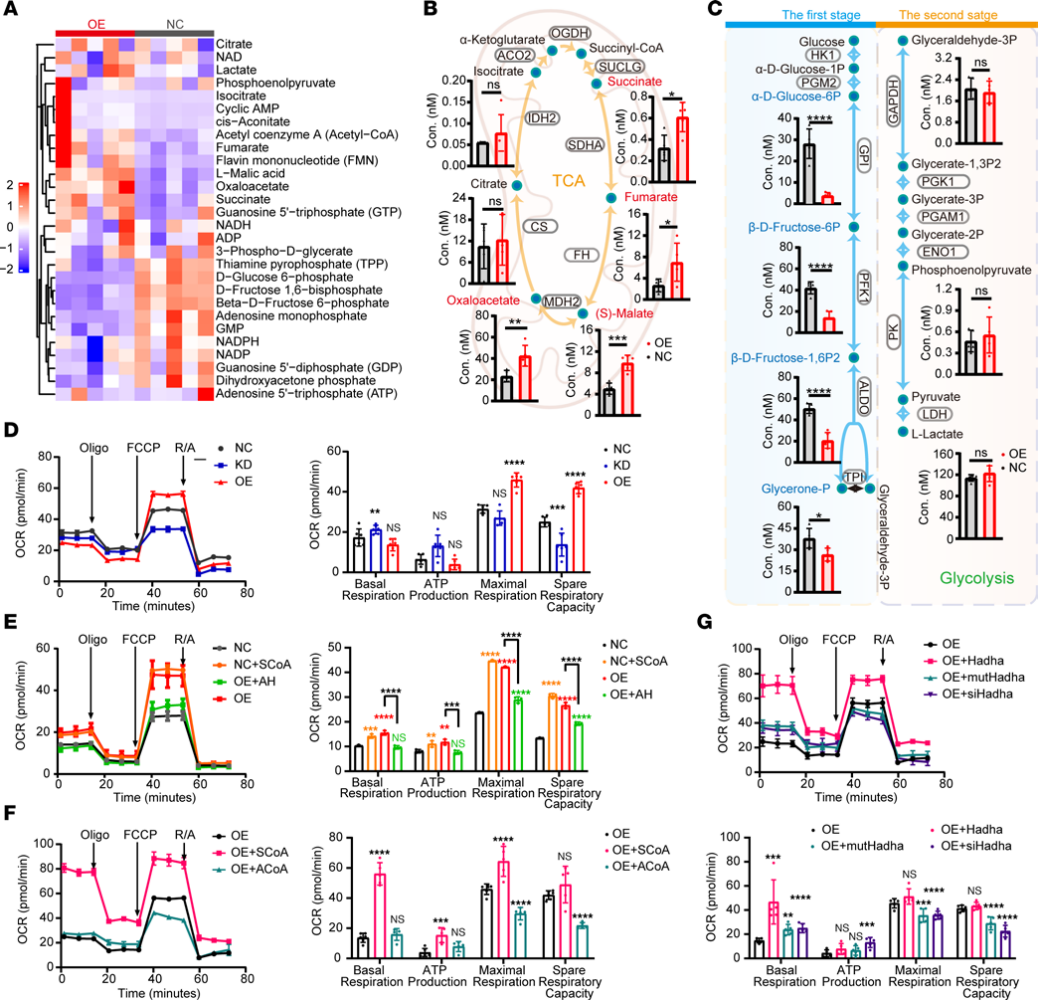
ACAT1 changes oxidative metabolism of cells through succinylation. (**A**–**C**) Targeted metabolic assays of *ACAT1* overexpression (OE) and negative control (NC) in HEK293T cells, *n* = 5 per group. Heatmap representing metabolite changes in OE compared with NC cells (**A**). Content differences of key metabolites in TCA (**B**). Content differences of key metabolites in glycolysis (**C**). (**D**–**G**) The oxygen consumption rate measurement of specific cells with indicated treatment: *Acat1* OE, NC, and knockdown (KD) Lewis lung carcinoma (LLC) cells, *n* = 6 per group (**D**); NC LLC cells, NC LLC cells pretreated with Succinyl-CoA (SCoA), OE LLC cells, OE LLC cells pretreated with ACAT1 inhibitor AH, *n* = 3 per group (**E**); OE LLC cells, OE LLC cells pretreated with Succinyl-CoA or Acetyl-CoA (ACoA), *n* = 6 per group (**F**); OE LLC cells, OE LLC cells with overexpression of *Hadha* or K305R mutant HADHA (mut*Hadha*), OE LLC cells with *Hadha* knockdown (si*Hadha*), *n* = 5 per group (**G**). Data are shown as the mean ± SD. *P < 0.05; **P < 0.01; ***P < 0.001; ****P < 0.0001 with 2-tailed unpaired Student’s *t* tests.

To assess whether ACAT1-mediated Ksucc affects cell metabolism, mass spectrometry–based targeted quantitative metabolomics detection was performed. We observed that the concentrations of some TCA cycle and glycolysis intermediates were significantly different between *ACAT1* NC and OE cell lines (Figure 6A and Supplemental Figure 6A). Acetyl-CoA utilized by CS is generally considered to initiate the TCA cycle pathway (8). Taking acetyl-CoA as the starting point of TCA, metabolites in the second half of TCA were upregulated in *ACAT1*-OE cell lines compared with those in the NC cell lines (Figure 6B). Interestingly, succinate and fumarate, 2 metabolites that have been identified as oncometabolites (33, 34), were increased (Figure 6B). By contrast, the concentrations of intermediates in the first stage of glycolysis were downregulated in *ACAT1*-OE cell lines compared with those in the NC cell lines (Figure 6C). In addition, the protein levels of enzymes involved in TCA cycle and glycolysis remained unaltered upon *ACAT1* expression, indicating that ACAT1 may transform cell metabolism, mainly by influencing protein posttranslational modifications (Supplemental Figure 6, B and C).

As ACAT1 is located in the mitochondria, we sought to study the role of ACAT1-mediated Ksucc in mitochondrial function. To better mimic the metabolic fate of lung cancer, stable *Acat1* KD/NC/OE LLC cell lines were used to study oxygen consumption rate (OCR) (Supplemental Figure 2, A and B). Our findings demonstrated that *Acat1* OE was linked to significantly higher maximal respiration and stronger spare-respiratory capacity, whereas *Acat1* KD resulted in weaker spare-respiratory capacity (Figure 6D). When supplementing the medium with the succinylation substrate SCoA before performing seahorse OCR tests, the broken line of NC cells representing OCR presented a consistent trend with OE cells, while arecoline hydrobromide (AH), a covalent ACAT1 inhibitor (14), counteracted the partial effect of OCR increase due to *Acat1* OE and dragged the OCR of OE cells to almost the same level of NC cells (Figure 6E). In addition, presupplementation of the medium with the acetylation substrate acetyl-CoA did not improve OCR function, as SCoA did (Figure 6F). When the succinylation site K305 of HADHA was mutated, OCR function was inhibited compared with normal HADHA-OE cells (Figure 6G). Overall, these results indicated that *Acat1* OE enhanced cell OCR through ACAT1-mediated succinylation instead of acetylation.

### Reducing oxidative stress triggered by ACAT1 facilitates B cell aggregation and TLS formation.

**Figure 7:**
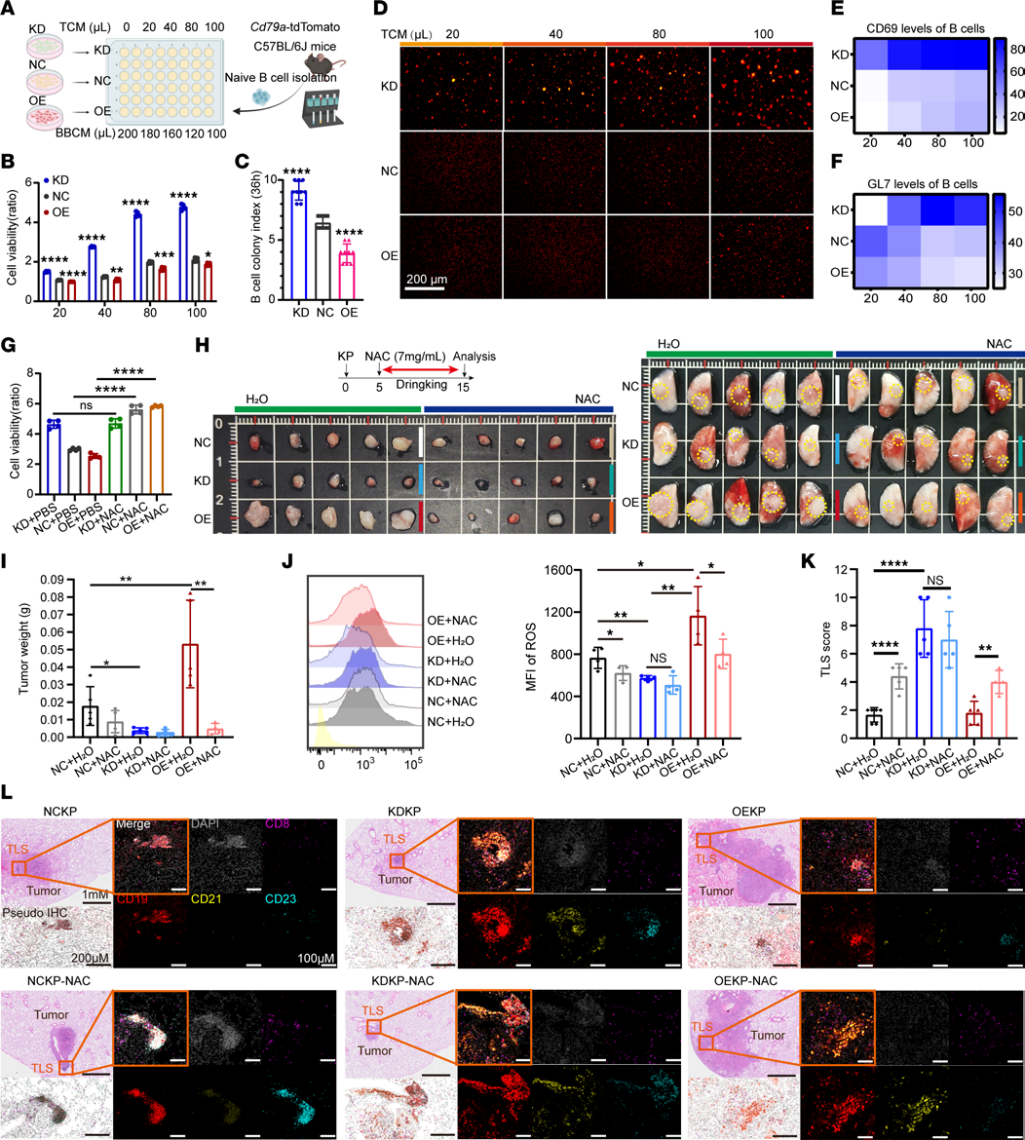
Reduction of oxidative stress promotes B cell aggregation and increases tertiary lymphoid structure abundance. (**A**) B cell culture in vitro with indicated tumor culture medium (TCM) from *Acat1* knockdown (KD)/negative control (NC)/overexpression (OE) LLC cells. BBCM, basic B cell culture medium. (**B**) Viability of B cells cultured in TCM at 36 hours determined by a CCK8 assay, *n* = 4 per group. (**C** and **D**) Representative images of B cell clumps cultured under TCM culture conditions, obtained by fluorescence microscopy, *n* = 9 per group (**C**). The B cell colony index was determined by the number and size of B cell colonies observed under microscope (**D**). (**E** and **F**) CD69 (**E**) and GL7 (**F**) levels of B cells cultured in TCM were determined by flow cytometry. (**G**) Viability of B cells cultured in TCM with or without NAC appending at 36 hours determined by a CCK8 assay, *n* = 4 per group. (**H**–**J**) NAC administered in drinking water was used to eliminate ROS in the KP models, followed by determination of tumor volume (**H** and **I**) and ROS clear effect (Tumor cells were labeled with DCFH-DA and detected by flow cytometry) (**J**). (**K** and **L**) Tertiary lymphoid structures (TLS) in mouse lung tissues of KP model were observed using H&E and immunofluorescence. Purple. CD8; red, CD19; yellow, CD21; blue, CD23; and gray, DAPI for nucleus. Representative staining images are shown (**L**). TLS score was determined by the number and maturity of TLS and the ratio of the total area of all TLS to the total tumor area, *n* = 5 per group (**K**). Data are representative of at least 2 independent experiments. Data are shown as the mean ± SD. *P < 0.05; **P < 0.01; ***P < 0.001; ****P < 0.0001 with 2-tailed unpaired Student’s t tests. Please also see Supplemental Figure 7.

To mimic TLS formation in the TME in vitro, we collected tumor culture medium (TCM) from KD, NC, and OE LLC cells, then constructed conditional culture medium (CM) for splenic B cell culturing by appending dosage-dependent TCM to the basal B cell culture medium (Figure 7A). B cell viability in the KD group was tested using CCK8 and found to be significantly more enhanced than that of the NC group, whereas it was attenuated in the OE group (Figure 7B). *Cd79a*-tdTomato mice that selectively fate map B cells following expression of the *CD79a* were generated (Supplemental Figure 7A), splenic B cells were isolated from them and cultured with CM, and B cell colony formation was observed under a fluorescence microscope. Both the number and size of B cell colonies increased with the dose of supplemented TCM, whereas the colony index of B cell aggregation was significantly decreased in the OE and increased in the KD group compared with the NC group, at the same time and dose (Figure 7, C and D). In addition, flow cytometry revealed that the expression level of B cell early activation antigen CD69 increased with the ratio of TCM to CM, while B cells in the KD group expressed the highest level of CD69 among all dose groups (Figure 7E and Supplemental Figure 7B). Meanwhile, the level of GL7, a germinal center marker, increased in line with the increased ratio of TCM in the KD group, whereas the expression trend was opposite in the NC and OE groups (Figure 7F and Supplemental Figure 7C). Overall, these results indicated that TCM affects B cell activation and viability, thereby influencing B cell aggregation in the TME.

Previous research has recognized that highly proliferative lung cancer cells exhibit higher OCR and elevated ROS (35). GSEA showed that the negative regulation of ROS biosynthesis was enriched in the KD group in our RNA-seq data (Supplemental Figure 7D). The oxidative stress levels in OE LLC cells were significantly higher than those in NC LLC cells, as confirmed by flow cytometry (Supplemental Figure 7E). To explore the relationship between ROS and TLS formation, we replenished the ROS scavenger N-acetylcysteine (NAC) with CM and observed that B cell viability recovered in the NC and OE groups (Figure 7G).

Experimental evidence indicates that NAC administered in vivo has a therapeutic effect. Both NC and OE tumor-bearing mice had a lower tumor burden after NAC supplementation in both the KP and LLC models Figure 7, H and I and Supplemental Figure 7, F and G). NAC eliminated ROS in NC and OE LLC tumor tissues (Figure 7J and Supplemental Figure 7, H and I). Interestingly, the increase in TLS formation appeared in both the NC and OE groups after NAC administration (Figure 7, K and L and Supplemental Figure 7, L and M). In addition, *Acat1* KD resulted in a decreased baseline level of ROS; NAC treatment thereafter failed to further augment the TLS score and decrease tumor burden in mice. In summary, *Acat1* overexpression in KP/LLC cells hindered TLS formation, and ROS scavenging recovers TLS formation impeded by ACAT1.

## Discussion

TLS formed by B cell aggregates are considered a hallmark of hot tumors and are associated with a favorable prognosis in 26 out of 31 cancer types in TCGA (36–38). Although the overlapping metabolic reprogramming of cancer and immune cells determines the antitumor immune response, how metabolic reprogramming of tumor cells regulates TLS and possible approaches to targeting metabolic pathways in the context of antitumor immunotherapy have been poorly studied. Here, using in vivo CRISPR screening, we found that ACAT1 linked tumor cell metabolism to TLS abundance in NSCLC. *Acat1* KD reduced the environmental stress caused by oxidative metabolism of tumor cells, thereby increasing TLS and tumor immunotherapy responsiveness.

ACAT1 is a mitochondrial enzyme with acetyltransferase activity involved in the ketone body metabolism, fatty acid β oxidation, and isoleucine degradation (39, 40). While previous studies have highlighted its oncogenic role in promoting tumor growth directly (14, 40), our findings reveal a novel mechanism by which ACAT1 impairs TLS formation, thereby facilitating tumor progression. Specifically, we found that ACAT1 acts as a potential mLSTase, which affected the Ksucc level of mitochondrial metabolism–related enzymes and enhanced the mitochondrial oxidative metabolism of NSCLC, subsequently blocking the formation of TLS in a ROS-dependent manner, and promoting tumor progression.

Overall metabolism reprogramming of tumors is often accompanied by extensive protein posttranslational modifications (PTMs), including protein succinylation, an evolutionarily conserved PTM where a succinyl group is mainly covalently bound to lysine residues. Ksucc induces more substantial changes in protein function compared with methylation or acetylation (41). SCoA, the critical metabolic intermediate in the TCA cycle, donates the succinyl group (42–45). Studies have revealed that Ksucc acts as a double-edged sword in tumorigenesis. For instance, K123succ of Cu/Zn superoxide dismutase (SOD1) promotes tumorigenesis in lung carcinoma (46), while SIRT5-mediated desuccinylation enhances tumorigenesis through inhibiting PKM2 (47). In our study, ACAT1 positively correlated with mitochondrial hypersuccinylation, and mitochondrial hypersuccinylation may be a satellite phenomenon in tumor progression.

Mitochondrial ROS (mtROS), primarily generated by the electron transport chain (ETC) and oxidative phosphorylation (OXPHOS), mediate various aspects of tumorigenesis, including proliferation, migration/invasion, angiogenesis, inflammation, and immune evasion (48, 49). Tumor cells use mtROS to induce immune tolerance, establishing an immunosuppressive TME. For example, the mitochondrial Lon-induced mtROS-NF-κB axis stimulates inflammatory cytokine release, contributing to immune suppression (50). Additionally, ROS can influence the expression of PD-1 and PD-L1 (51). By focusing on how metabolism and redox status influence the interplay between cancer cells and immune cell infiltration, we provided direct evidence that high oxidative stress mediated by ACAT1 retarded TLS formation in the TME.

Single-cell sequencing and flow cytometry analysis showed that *Acat1*-KD tumors had reduced monocyte cells and increased CD4^+^ but not CD8^+^ T cells. The increased CD4^+^ T cells may be helpful in assisting TLS formation, as shown in previous studies (52). The CD8^+^ T cells did not increase, suggesting that antibody-dependent cellular cytotoxicity (ADCC) independent of T cell killing contributed to the antitumor efficacy. Additionally, *Acat1* KD may indirectly enhance antitumor immune efficacy by decreasing the proportion of monocyte-derived tumor-associated macrophages (TAMs), tumor-associated DCs (TADCs), and myeloid-derived suppressor cells (MDSCs) in the TME, all of which primarily mediate an immunosuppressive microenvironment (53).

In both mice and humans, the TLS formed in response to pulmonary inflammation within lung tissue is also designated as induced bronchus-associated lymphoid tissue (iBALT) and can rarely be observed under physiological status (54, 55). Similarly, we confirmed that TLS structures were induced by tumors, because they were not present in normal lung tissue distant from tumor regions in tumor-bearing KP mice, nor in lung tissue of healthy mice without tumor. An important site for the formation of TLS/iBALT is the perivascular space, which is typically parallel to bronchial airways; therefore, the development of TLS/iBALT also tends to occur in proximity to the airways (55).

In summary, our results revealed how cancer-cell metabolic reprogramming regulates the antitumor immune response and identified ACAT1 as an important metabolic regulator for TLS-related antitumor immunity. ACAT1 mediates the hypersuccinylation of mitochondrial proteins in lung tumor cells and subsequently enhanced mitochondrial oxidative metabolism, which impedes TLS formation and antitumor immunity. *Acat1* KD improved B cell and TLS abundance and increased the sensitivity of lung cancer cells to anti-PD1 immunotherapy. Given the value of TLS in predicting tumor progression and immunotherapy response, ACAT1, a metabolic regulator of TLS, is a promising immunotherapeutic target in NSCLC.

## Methods

### Sex as a biological variable.

Our study examined male and female animals, and similar findings are reported for both sexes.

Additional details can be found in the Supplemental Methods.

### Patient specimens.

All NSCLC patient samples were obtained from patients being treated at the Shanghai Zhongshan Hospital. Among these, the lung cancer tissue microarray including 305 patients was derived from the Zhongshan Hospital cohort (ZSNH cohort). Tumor puncture samples of 29 patients with NSCLC who were treated with immunotherapy were derived from the Immunotherapy cohort (ICB cohort) (16). The detailed clinical and pathologic information for the patients is listed in Supplemental Table 4. All prognostic studies followed the REMARK (REporting recommendations for tumor MARKer prognostic studies) reporting guidelines, and the description of the specimens was carried out per the BRISQ (Biospecimen Reporting for Improved Study Quality) reporting guidelines.

### Mice.

C57BL/6, *Rag*^–/–^, and C57BL/6-*ROSA^26^CAG-tdTomato* mice were obtained from Gempharmatech Co. Ltd. (Jiangsu, China). *Cd79a*-cre mice were a gift from Zhongjun Dong (Tsinghua University, Beijing, China). *ROSA^26^CAG-tdTomato* mice were respectively crossed with *Cd79a*-cre transgenic mice to generate B cell reporter mice (*CD79a*-tdTomato mice), *CD79a*-tdTomato mice were genotyped and selected for experiments. All the mice used were housed in the animal facility of Fudan University (Shanghai, China) under specific pathogen-free conditions. *Acat1*^–/–^ mice were derived from C57BL/6 strain, and provided by Cyagen. All the mice used were housed in the animal facility of Fudan University (Shanghai, China) under specific pathogen-free conditions and were used for the described experiments at 8–10 weeks of age. The details of genotyping were shown in Supplemental Table 5.

### Cell lines.

HEK293T cells and Lewis lung carcinoma (LLC) cells were obtained from American Type Culture Collection. Murine KrasG12D/p53^−/−^ (KP) lung cancer cell line (C57BL/6 background) and KP-Cas9 cells were established and characterized as described previously (20). HEK293T cells were cultured in DMEM containing 10% FBS and 1% penicillin-streptomycin. LLC cells, KP cells, and KP-Cas9 cells were cultured in RPMI-1640 medium containing 10% FBS and 1% penicillin-streptomycin. ACAT1 control/overexpression/knockdown (NC/OE/KD) LLC and KP cell lines were constructed using a lentivirus vector synthesized by Shanghai Genechem Co. Ltd. according to the manufacturer’s instructions as previously described (56). Cells were grown at 37°C in a 5% CO_2_ setting.

### Data acquisition from TCGA and gene expression analysis.

LUAD gene expression data and survival information were obtained from TCGA database. TCGA raw RNA-seq counts and corresponding clinical data were downloaded using the R package TCGAbiolinks (57). A total of 600 samples, including 533 LUAD and 67 normal thyroid tissue samples, were collected. Grouping of 533 tumor tissue samples was performed according to TSGs expression levels using the Rstudio version 4.3.2 and Bioconductor R packages, especially “gsva”. To investigate the biological functions and key pathways associated with TLS formation in LUAD, we conducted GO (58) and KEGG (59) enrichment analyses using R packages org.hs.eg.db, DESeq2 (60), clusterProfiler (61), and ggplot2. Statistical significance was set at a *P* < 0.05 level.

### Construction of PPI network and identification of metabolic hub genes.

To characterize protein-protein interactions (PPIs) between the upregulated metabolic genes in the TLS_low group, we constructed a PPI network using the STRING database version 12.0 (13). Genes with a minimum required interaction score of 0.5 were selected from the database to build a full network model, which was visualized using Cytoscape version 3.10.1 (62). Hub genes in the network were identified using the MCC algorithm implemented in the cytoHubba Cytoscape plugin, which retained the top ten hub genes. The network was then visualized using Cytoscape to depict the hub gene interactions. PPI network analysis provided insights into the key proteins and modules involved in TLS formation.

### Orthotopic lung tumor models.

Orthotopic transplantation of tumor cells was performed as previously described (24, 25). Briefly, 3.5 × 10^5^ LLC/KP/KP-Cas9 cells embedded in Matrigel (BD Biosciences) were injected in 15 μL aliquots into the left lung of mice between the fifth and seventh ribs, using a 30-gauge needle attached to a 50-μL Hamilton syringe (Hamilton Company, Switzerland). The mice were monitored for 1 hour after injection to ensure recovery and humanely euthanized 15 days after tumor cell transplantation to harvest the lungs. Tumors were separated from mouse lungs, and tumor volumes were calculated using the formula:
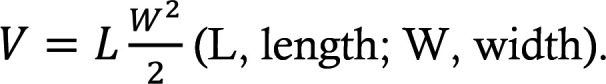


### In vivo CRISPR Screening for metabolic regulators of immunotherapy.

To determine how tumor propagation in vivo affects basal sgRNA library distribution, clones without Cas9 expression were transduced at a low multiplicity of injection (MOI) with the sgRNA library for use as barcodes. Following several days of expansion in medium with 5 μg/mL blasticidin (Yeasen, China), a pellet of at least 8 million cells was kept to evaluate the initial sgRNA distribution, and the remainder was injected orthotopically into *Rag1*^−/−^ and C57BL/6 mice. The tumors were allowed to grow 14 days and then excised for NGS analysis to evaluate the final library distribution. For the functional screening, LLC-Cas9 cells were generated by viral transduction and Cas9 expression was confirmed by Western blotting. LLC-Cas9 clones with validated Cas9 activity were transduced at a MOI of 0.3 with lentivirus produced from the libraries with at least 1,000-fold coverage (cells per construct) in each infection replicate. Transduced LLC cells were expanded in vitro for 2 weeks and then subcutaneously implanted into *Rag1*^−/−^ and C57BL/6 mice. The mice were then treated with anti-PD1 or an isotype control on days 7 and 11, and the tumors were harvested on day 15. Genomic DNA from tumors or cells was extracted using a DNA Blood Midi Kit (Qiagen), and sgRNA abundance was determined by NGS after PCR amplification of the sgRNA cassette on an Illumina TruSeq (63, 64). The manipulation of NGS was conducted in GenePlus (Beijing, China).

### Immunofluorescence and IHC examination.

IF and IHC staining were performed as previously described (65). Briefly, paraffin-embedded sections were deparaffinized and subjected to antigen retrieval and blocking of nonspecific binding. For multiplex IF, sections were incubated with primary antibodies against CD8 (1:100, ab101500, Abcam), CD19 (1:100, no. 90176, Cell Signaling Technology), CD21 (1:100, ab75985 and ab227662, Abcam), and CD23 (1:100, ab92495 and ab315289, Abcam), followed by the manual of OpalTM 7-Color Manual IHC Kit (PerkinElmer), and analyzed by TissueFAXS Cytometry (TissueGnostics, USA). For IHC, sections were incubated with an anti-ACAT1 antibody (1:100, no. 44276, Cell Signaling Technology) in phosphate-buffered saline with 3% BSA, followed by horseradish peroxidase–conjugated secondary antibodies according to the manual of the SABC-HRP Kit (P0615, Beyotime), and analyzed by Olympus Slideview VS200.

### Identification of TLS and ACAT1 IHC levels in NSCLC.

TLS and ACAT1 levels in lung cancer samples were quantified by 2 independent lung cancer–specialized pathologists who were blinded to patients’ clinical and molecular characteristics. TLS were assessed on H&E-staining and IF-staining slides based on dense lymphocyte aggregates. Tumors containing at least 1 intratumoral TLS were designated TLS positive (TLS+), while TLS-negative (TLS–) tumors lacked any TLS. Accordingly, the maturation state of each TLS was subsequently classified as eTLS, pTLS, or sTLS based on the presence of a CD21^+^ follicular dendritic cell (FDC) network (pTLS), a CD23^+^ germinal center (sTLS), or the absence of these features (eTLS). TLS scoring system containing the number of TLS near the tumor, the area density of TLS (TLS area/Tumor area), and the maturity of TLS (eTLS, pTLS, sTLS), as previously described (10, 18). ACAT1 IHC scores ranged from 0 to 3 based on staining intensity: 0 = no signal, 1 = weak, 2 = moderate, and 3 = strong. Scores of 0–1 and 2–3 were classified as low (ACAT1^Lo^) and high (ACAT1^Hi^) expression, respectively.

### Isolation of immune cells from lung tissues.

Lung tumors were minced into 1 mm^3^ sections and digested for 45 minutes at 37°C in RPMI-1640 medium containing 10% FBS, 1 mg/mL collagenase D (Roche Applied Science), and 0.08 mg/mL DNase I (Sigma-Aldrich). The cell suspension was filtered through a 70-μm nylon cell strainer (Corning) and collected. Immune cells were isolated from lung tissue by centrifugation over 40% discontinuous Percoll gradients and used for flow cytometry analysis. Cells were stained by fluorochrome-conjugated antibodies, consistent with previous procedures (66).

### Immunoprecipitation.

Cells were lysed in RIPA buffer (Beyotime Biotechnology) supplemented with protease inhibitors (Beyotime Biotechnology). For immunoprecipitation (IP), cell lysates were incubated overnight at 4°C with the indicated primary antibodies, followed by addition of Protein A/G Agarose Beads (Roche) for 1 hour. Bead complexes were washed 3 times with RIPA buffer before analyzed by immunoblotting.

### In vitro succinylation assays.

In vitro succinylation assays were performed as described previously (31, 67). Full-length His-ACAT1 and His-HADHA were expressed in HEK293T cells. Soluble His-tagged proteins were purified using Ni-NTA smart beads (Smart-Lifesciences) according to the manufacturer’s instructions. To analyze ACAT1-mediated HADHA succinylation, purified His-HADHA was incubated with active His-ACAT1 or heat-inactivated His-ACAT1 (boiled at 100°C for 30 minutes) in TBS buffer containing 2 μM succinyl-CoA at 37°C for 10 minutes. HADHA succinylation levels were assessed by immunoblotting.

### Seahorse analysis.

Oxygen consumption rates (OCR) were measured as previously described using an XF96 Extracellular Flux Analyzer (Agilent) (66). Cells were plated 1 day prior to the assay and basal OCR was measured, followed by sequential injections of 1 μM oligomycin (Complex V inhibitor), 2.0 μM FCCP (uncoupler), and 0.5 μM rotenone/antimycin A (Complex I/III inhibitors) using the Mito Stress Test kit (Agilent).

### Targeted metabolomics.

Cells (1 × 10^7^) were dry frozen in liquid nitrogen and then thawed at 4°C and mixed with 1 mL of cold methanol/acetonitrile/H2O (2:2:1, v/v/v). The homogenate was sonicated at low temperature (30 minutes, 2 times), The mixture was centrifuged for 20 minutes (14000*g*, 4°C). The supernatant was dried in a vacuum centrifuge. For LC-MS analysis, the samples were redissolved in 100 μL acetonitrile/water (1:1, v/v) and adequately vortexed, and then centrifuged (14,000*g*, 4°C, 15 minutes). The supernatants were collected for LC-MS/MS analysis. Analyses were performed using an UHPLC (1290 Infinity LC, Agilent Technologies) coupled to a QTRAP (AB Sciex 5500). LC-MS quantification of energy metabolites achieved with 6-point standard curves using SUCCINIC ACID (D6, 98%, DLM-831-5, CIL) diluted in a relevant matrix matched to the analytical sample (absolute quantification). Data acquisition and processing were accomplished using MultiQuant software. The manipulation was conducted in Shanghai Applied Protein Technology Co. Ltd.

### B cell culture in vitro.

To obtain TCM, 2.5 × 10^6^ KD/NC/OE LLC cells with 5 mL RPMI-1640 medium containing 10% FBS and 1% penicillin-streptomycin were initially planked in 6-cm cell culture dishes (Corning) and cultured for 24 hours, then replaced with 5 mL RPMI-1640 medium and cultured for another 24 hours. The final number of cells were recorded and the entire TCM was collected separately. After removed cell debris by centrifugation (3000*g*, 1 minute) and filtration (0.22 um, Sigma-Aldrich), the TCM was flash frozen in liquid nitrogen for 15 minutes and then transferred to –80°C for storage. We constructed CM utilized for splenic B cell culturing by appending dosage dependent TCM to the RPMI-1640 medium, 10% FBS, 1% penicillin-streptomycin. For B cell in vitro experiments, bulk B cells were purified from spleen using B cell isolation kit (purity greater-than 90%, STEMCELL). These purified B cells were cultured in CM and grown at 37°C in a 5% CO_2_ setting for 36 hours or the indicated times at the experimental endpoint.

### Statistics.

Statistical analyses were performed using GraphPad Prism 9 (GraphPad Software). Comparisons between 2 groups were made using an unpaired 2-tailed Student’s *t* test. Flow cytometry data are presented as mean ± SD. Kaplan-Meier analysis with log-rank tests was used to evaluate differences in survival. Univariate and multivariate Cox proportional hazards regression modeling was used. Statistical details are provided in corresponding figure legends. *P* < 0.05 was considered significant.

### Study approval.

Written informed consent was obtained from each patient, and the study was approved by the Institutional Review Board and Ethics Committee of Zhongshan Hospital, Fudan University. All animal experiments were carried out in accordance with the Guide for the Care and Use of Laboratory Animals and were approved by the Animal Studies Committee at Fudan University.

### Data availability.

NGS raw data for CRISPR screen have been deposited in the National Center for Biotechnology Information Sequence Read Archive and are accessible through accession number (PRJNA1091422). The mass spectrometry proteomics data have been deposited to the ProteomeXchange Consortium via the iProX partner repository (68) with the dataset identifier PXD045413. A Supporting Data Values file with all reported data values is available as part of the supplemental material. Complete unedited gel images are provided in the supplemental materials. Other sources of protein and nucleic acid sequences are indicated in the manuscript and are available from the corresponding author on request.

## Author contributions

MJ, YG, and HZ performed the research, discussed and analyzed the data, and wrote the paper. YC, JG, RL conceived of and supervised the study, designed the research, interpreted the results, and wrote the paper. HW, PC, ZW, BY, YG, KZ, YZ, JQ, YX, PZ, ZP, LW, DZ, YR, CC assisted experiment and provided reagents or technical support FL guided the construction of the KP model. RL is the primary contact for communication.

## Supplementary Material

Supplemental data

Unedited blot and gel images

Supplemental table 1

Supplemental table 2

Supplemental table 3

Supplemental table 4

Supplemental table 5

Supplemental table 6

Supporting data values

## Version 1 04/01/2025

Electronic publication
