# Supplementary material for: ACAT1 regulates tertiary lymphoid structures and correlates with immunotherapy response in non–small cell lung cancer
Source: J Clin Invest. 2025 Apr 1;135(7):e181517. doi: 10.1172/JCI181517 (PMC11957694; doi:10.1172/JCI181517)
Supplement: Supplemental data [file jci-135-181517-s153.pdf]

Supplemental information

ACAT1 regulates tertiary lymphoid structures and correlates with immunotherapy response in non-small-cell lung cancer

Author names

Mengxia Jiao, Yifan Guo, Hongyu Zhang, Haoyu Wen, Peng Chen, Zhiqiang Wang, Baichao Yu, Kameina Zhuma, Yuchen Zhang, Jingbo Qie, Yun Xing, Pengyuan Zhao, Zihe Pan, Luman Wang, Dan Zhang, Fei Li, Yijiu Ren, Chang Chen, Yiwei Chu, Jie Gu, Ronghua Liu

Method Details

Succinyl-CoA affinity screen and molecular docking analysis

The succinyl-CoA affinity screen was conducted in Python 3.9 based on molecular docking analysis. Crystal structures of protein isoforms and the ligand succinyl-CoA were obtained from the Protein Data Bank and ZINC databases. Ligand and receptor structures were pretreated using Meeko 0.2 and OpenBabel 2.3.2, and corrected to pH 7.4 prior to docking analyses. AutoDock Vina v1.2.0 was used to calculate ligand-protein binding affinities for each protein-succinyl-CoA pair. Grid boxes for docking were automatically designated using Autogrid4. An "affinity index" was defined as the reciprocal of the binding affinity to indicate probable interaction. An affinity index threshold of 4 was set to predict potential succinyl-transferase activity.

Docking of succinyl-CoA (PDB: 5TRL) to ACAT1 (PDB: 2F2S) was performed using AutoDock Vina with minor modifications to reported parameters(1). The ACAT1 and succinyl-CoA structures were downloaded from the Protein Data Bank. Protein-ligand interactions were analyzed using the PLIP(2) web server.

Mitochondria Isolation and immunoblotting

Mitochondria were isolated from cells or tumor samples using a mitochondrial isolation kit (Beyotime Biotechnology) according to the manufacturer's instructions. Protein concentration was determined by bicinchoninic acid assay (Beyotime Biotechnology). Equivalent amounts of mitochondrial protein lysates were boiled in 1× SDS loading buffer (Beyotime Biotechnology) for 10 minutes, separated by SDS-PAGE at 80 V for 30 minutes followed by 100 V for 1 hour, and transferred to polyvinylidene difluoride membranes (0.45 μm, Millipore) at 200 mA for 2 hours. Membranes were blocked with 5% bovine serum albumin for 1 hour before overnight incubation at 4°C with anti-succinyllysine antibodies (1:2000, #PTM-401, PTM Biosciences). Membranes were subsequently incubated with peroxidase-conjugated goat

anti-rabbit secondary antibody (Yeasen) and visualized using Super ECL Detection Reagent (Yeasen).

Single-cell sequencing

BD Rhapsody system was used to capture the transcriptomic information of the tumor-derived single cells. Single-cell capture was achieved by random distribution of a single-cell suspension across >200,000 microwells through a limited dilution approach. Beads with oligonucleotide barcodes were added to saturation so that a bead was paired with a cell in a microwell. The cells were lysed in the microwell to hybridize mRNA molecules to barcoded capture oligos on the beads. Beads were collected into a single tube for reverse transcription and ExoI digestion. Upon cDNA synthesis, each cDNA molecule was tagged on the 5′ end (that is, the 3′ end of a mRNA transcript) with a unique molecular identifier (UMI) and cell barcode indicating its cell of origin. Whole transcriptome libraries were prepared using the BD Rhapsody single-cell whole-transcriptome amplification (WTA) workflow including random priming and extension (RPE), RPE amplification PCR and WTA index PCR. The libraries were quantified using a High Sensitivity DNA chip (Agilent) on a Bioanalyzer 2200 and the Qubit High Sensitivity DNA assay (Thermo Fisher Scientific). Sequencing was performed by illumina sequencer (Illumina, San Diego, CA) on a 150 bp paired-end run. scRNA-seq data analysis was performed by NovelBio Bio-Pharm Technology Co.,Ltd. with NovelBrain Cloud Analysis Platform. We applied fastp(3) with default parameter filtering the adaptor sequence and removed the low-quality reads to achieve the clean data. UMI-tools(4) were applied for Single Cell Transcriptome Analysis to identify the cell barcode whitelist. The UMI-based clean data was mapped to mouse genome (GRCm39) utilizing STAR(5) mapping with customized parameter from UMI-tools standard pipeline to obtain the UMIs counts of each sample.

Supplemental Figures

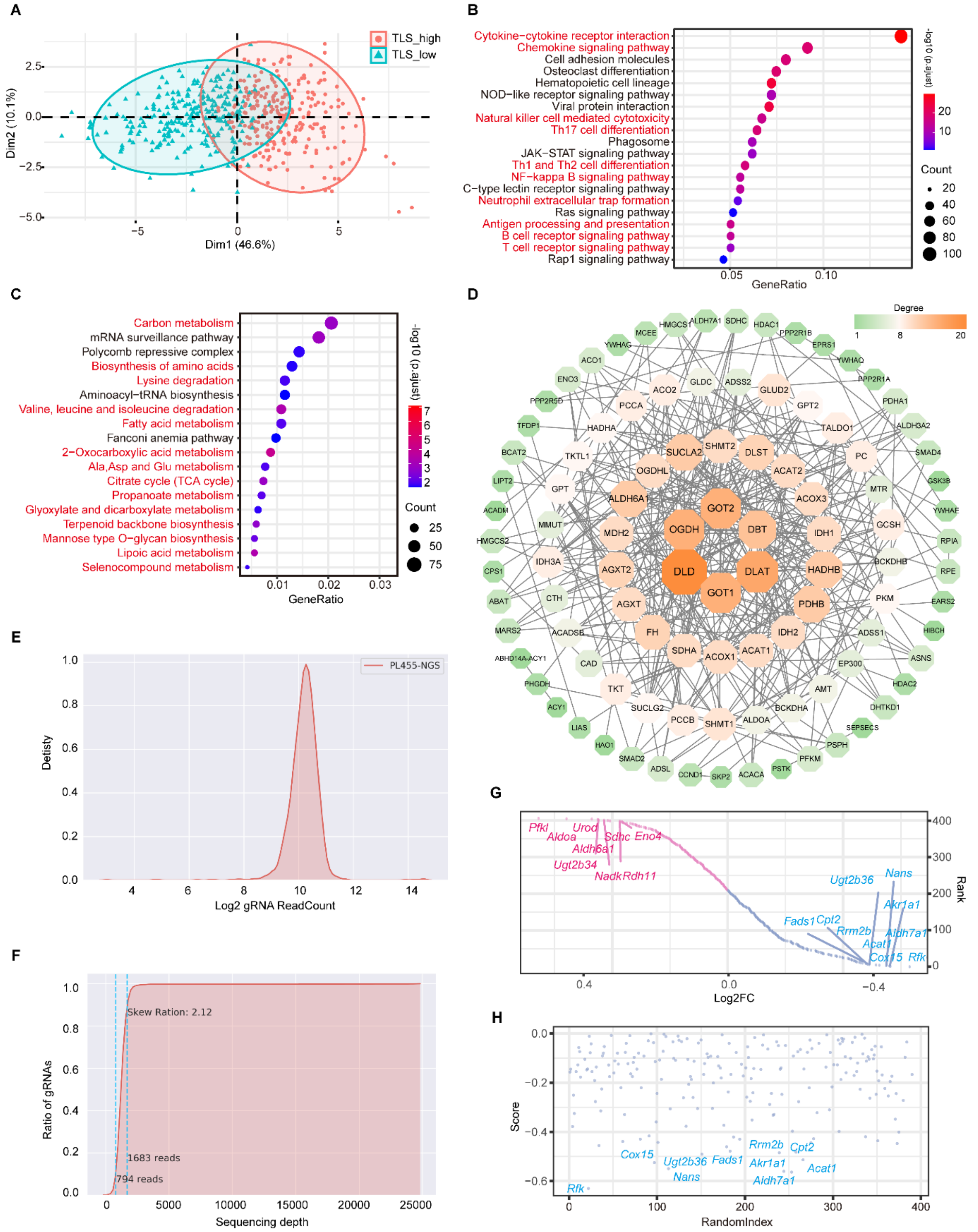

Figure S1. Identification of metabolic hub genes for tertiary lymphoid structure in non-small-cell lung cancer. (A) PCA of TLS_high group and TLS_low group. **(B)** Kyoto Encyclopedia of Genes and Genomes pathway enrichment analysis of genes with significant differences in terms of increase in TLS_high **(B)** or decrease in TLS_low **(C)** groups. **(D)** Search Tool for the Retrieval of Interacting Genes/Proteins database analysis for metabolic genes in **(C)**. **(E)** Kernel density estimation for gRNA Readcount, reflecting the distribution characteristics of the sgRNA. **(F)** Skew Ration of gRNA Distribution. Skew Ration: 2.12. It is generally believed that a Skew Ration of <10 signifies that the library is uniform. **(G)** Schematic representation of the top 10 candidates whose KO is expected to enhance (blue) or inhibit anti-PD1(red) treatment. RRA, Robust Rank Aggregation. **(H)** Illustration of the top-10 candidates whose knockout is expected to enhance anti-PD1 treatment in **(G)**.

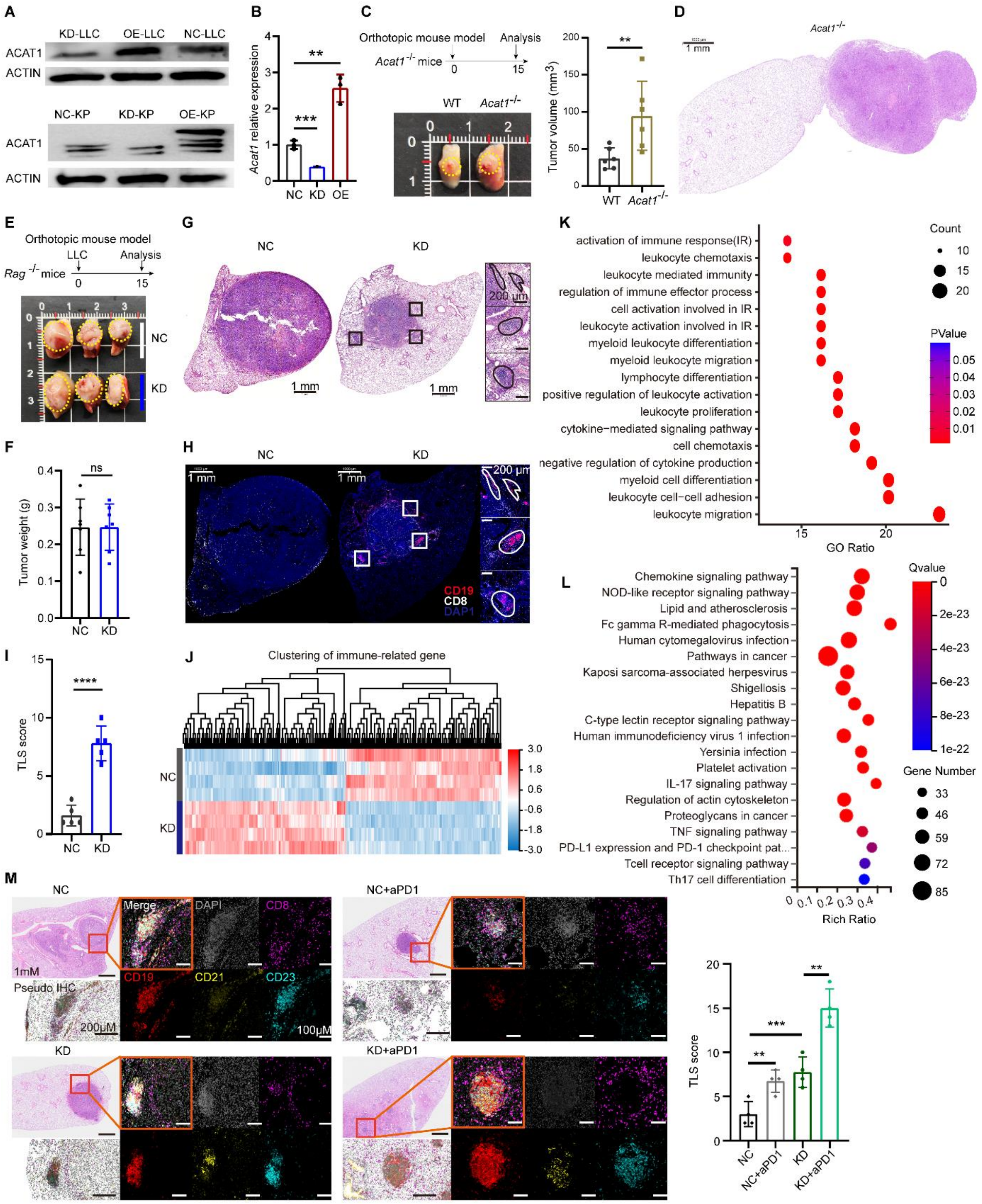

Figure S2. ***Acat1* knockdown reshapes the tumor microenvironment and effects anti-tumor immunity.** **(A)** ACAT1 expression measured as protein levels in knockdown (KD)/negative control (NC)/overexpressing (OE) Lewis lung carcinoma (LLC) and Kras^{G12D}/P53$^{-/-}$ (KP) cell lines. **(B)** ACAT1 expression mRNA levels in KD/NC/OE LLC cells. **(C)** Tumor development was measured in *Acat1*$^{-/-}$ LLC models, n = 6 per group. **(D)** H&E staining shows the tumor site and adjacent normal tissues of **(C)**. **(E-I)** Tumor development was measured in Rag-/- LLC models **(E)**. Tumor weight, n = 6 per group. **(F)**. Tertiary lymphoid structures (TLS) in mice lung tissues of LLC models were observed using H&E **(G)** and immunofluorescence **(H)**. Red: CD19; white: CD8; and blue: DAPI for nuclei. TLS score, n = 5 per group. **(I)**. **(J-L)** Gene cluster analysis showed immune-related gene expression level of NC and KD LLC cells identified from RNA-seq analysis **(J)**. **(K)** Kyoto Encyclopedia of Genes and Genomes pathway analysis for differentially expressed genes in (J). **(L)** Gene Ontology analysis in (J). **(M)** TLS in mice lung tissues of Figure 3F were observed using H&E and immunofluorescence, n = 4 per group. Purple: CD8; Red: CD19; yellow: CD21; Blue: CD23 and Grey: DAPI for nucleus. Representative staining images are shown. Data are shown as the mean ± SD. *, P < 0.05; **, P < 0.01; ***, P < 0.001; ****, P < 0.0001. ns, not significant, with 2-tailed unpaired Student's t tests.

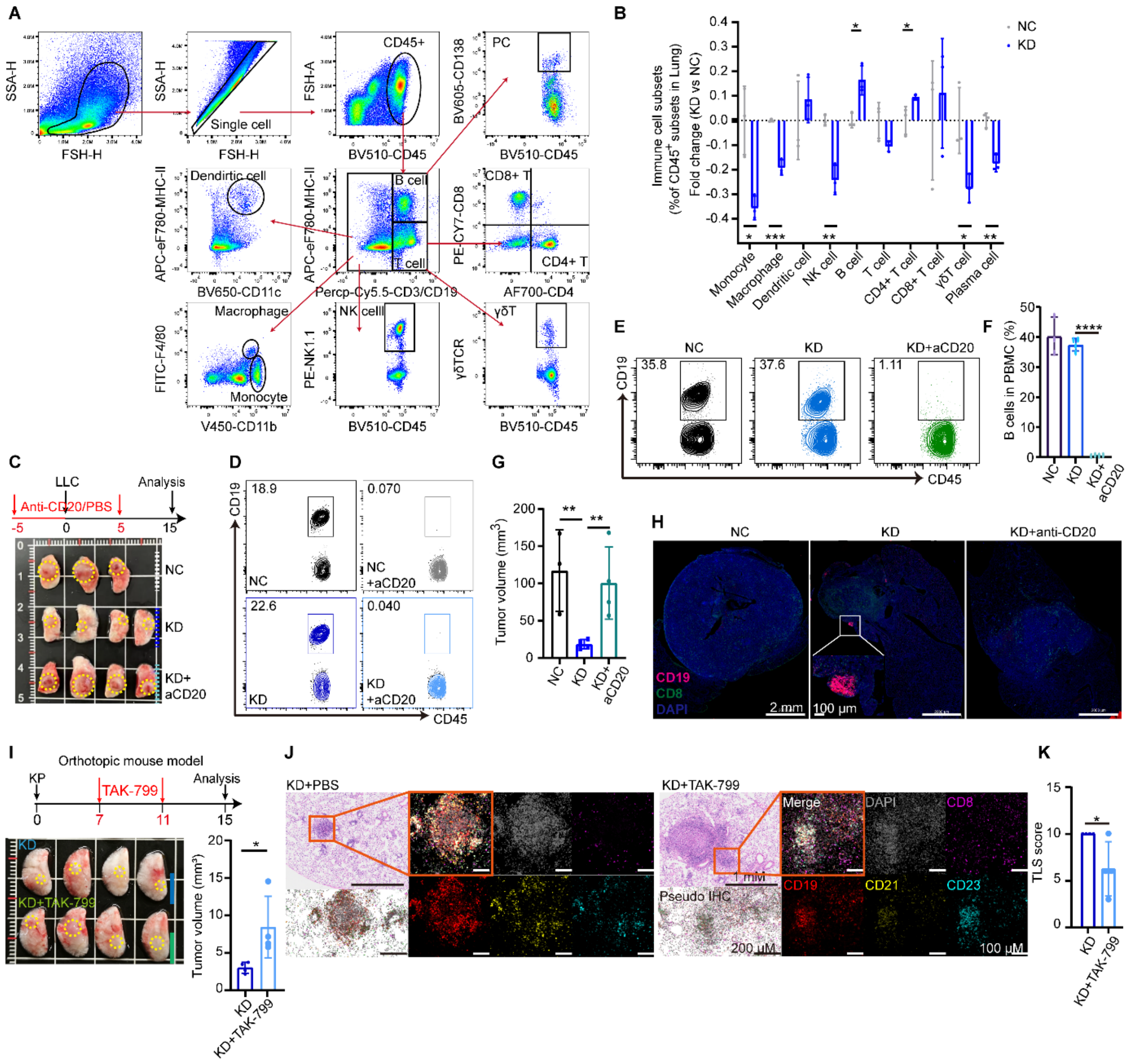

Figure S3. ***Acat1*** **knockdown enhances antitumor effect which relies on the formation of tertiary lymphoid structure harboring B cells. (A)** Gating strategy for the flow cytometry. **(B)**. The fold change in various immune cell subsets within mouse tumors was confirmed through flow cytometry, n = 3 per group. **(C)** Anti-CD20 antibodies were used to eliminate B cells in the orthotopic lung tumor mice model. **(D-F)** Flow cytometry was utilized to assess the depletion effect on B cells within the PBMCs. B-cell depletion effect in Figure 4J **(D).** B-cell depletion effect in (B) **(E-F). (G)** Tumor volume analysis of (B), n = 4 per group. **(H)** Tertiary lymphoid structure (TLS) in mouse lung tissues in (B) were observed by immunofluorescence. Red, CD19; green, CD8; and blue: DAPI for the nuclei. **(I)** TAK-799 was used to forbidden TLS formation in the orthotopic lung tumor mice model, n = 4 per group. **(J-K)**. TLS in mice lung tissues of (K) were observed using H&E and immunofluorescence**.** Purple: CD8; Red: CD19; yellow: CD21; Blue: CD23 and Grey: DAPI for nucleus. Representative staining images are shown **(I)**. TLS score was determined by the number and maturity of TLS as well as the ratio of the total area of all TLS to the total tumor area **(J)**. Data are representative of at least 2 independent experiments. Data are shown as the mean ± SD. *, P < 0.05; **, P < 0.01; ***, P < 0.001; ****, P < 0.0001. ns, not significant, with 2-tailed unpaired Student's t tests.

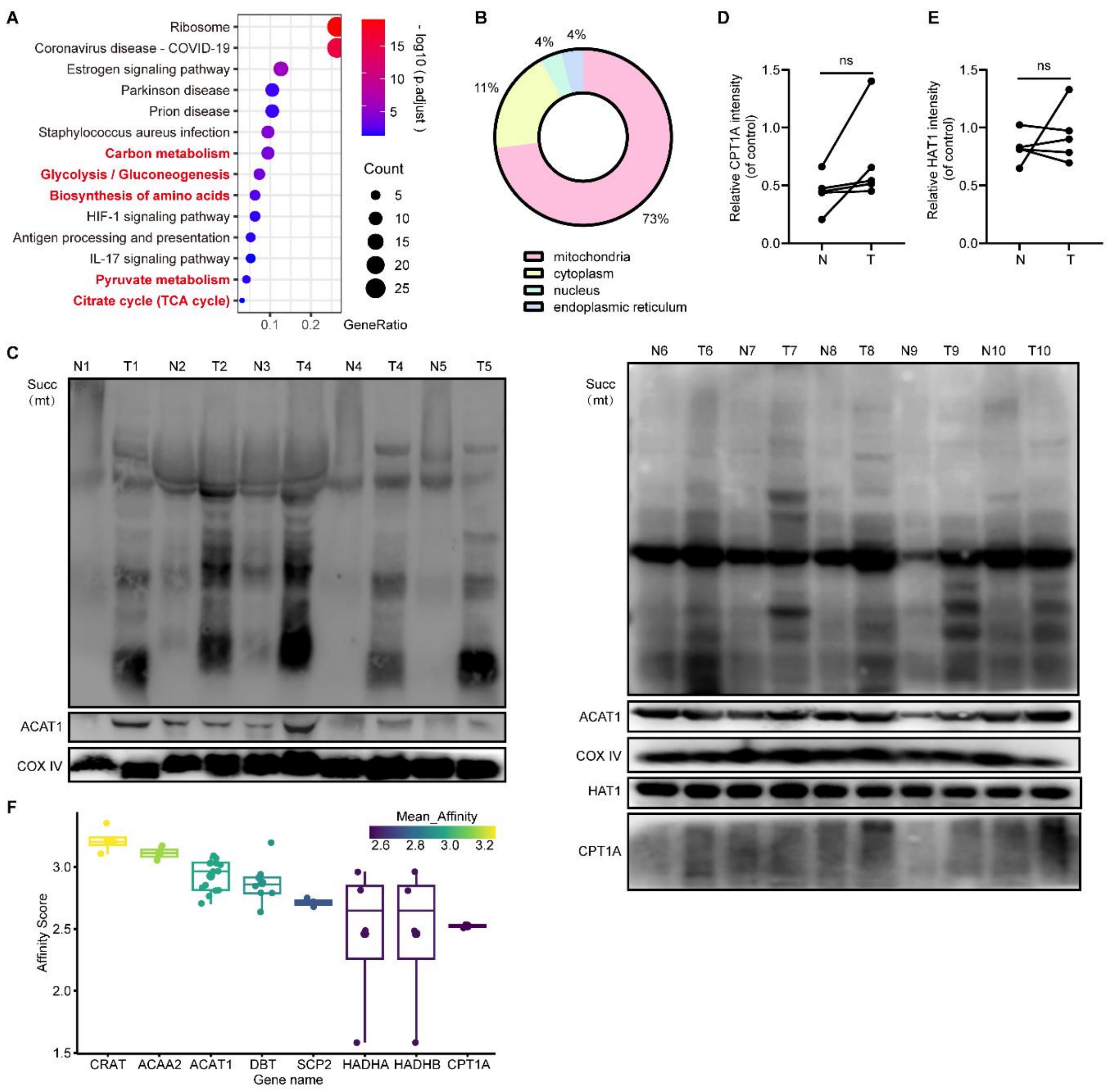

Figure S4. ACAT1 is correlated with mitochondrial protein hypersuccinylation in human lung cancer. **(A)** Kyoto Encyclopedia of Genes and Genomes pathway analysis for target proteins of ACAT1 detected by ACAT1-IP. **(B)** Distribution of succinylation (Succ) levels in cellular compartments. **(C-E)** Mitochondria were extracted from carcinoma (T) and adjacent tissues (N) of patients with lung cancer, and mitochondrial lysates were prepared. The total mitochondrial protein succinylation levels were analyzed by IB with the indicated antibodies. A comparison of CPT1A **(D)** and HAT1 **(E)** levels between carcinoma (T) and adjacent tissue (N) of **(C)**. Paired Student's t tests**. (F)** Potential succinyltransferases identified by a succinyl-transferase screen of mitochondrial proteins based on affinity between target proteins and succinyl-CoA.

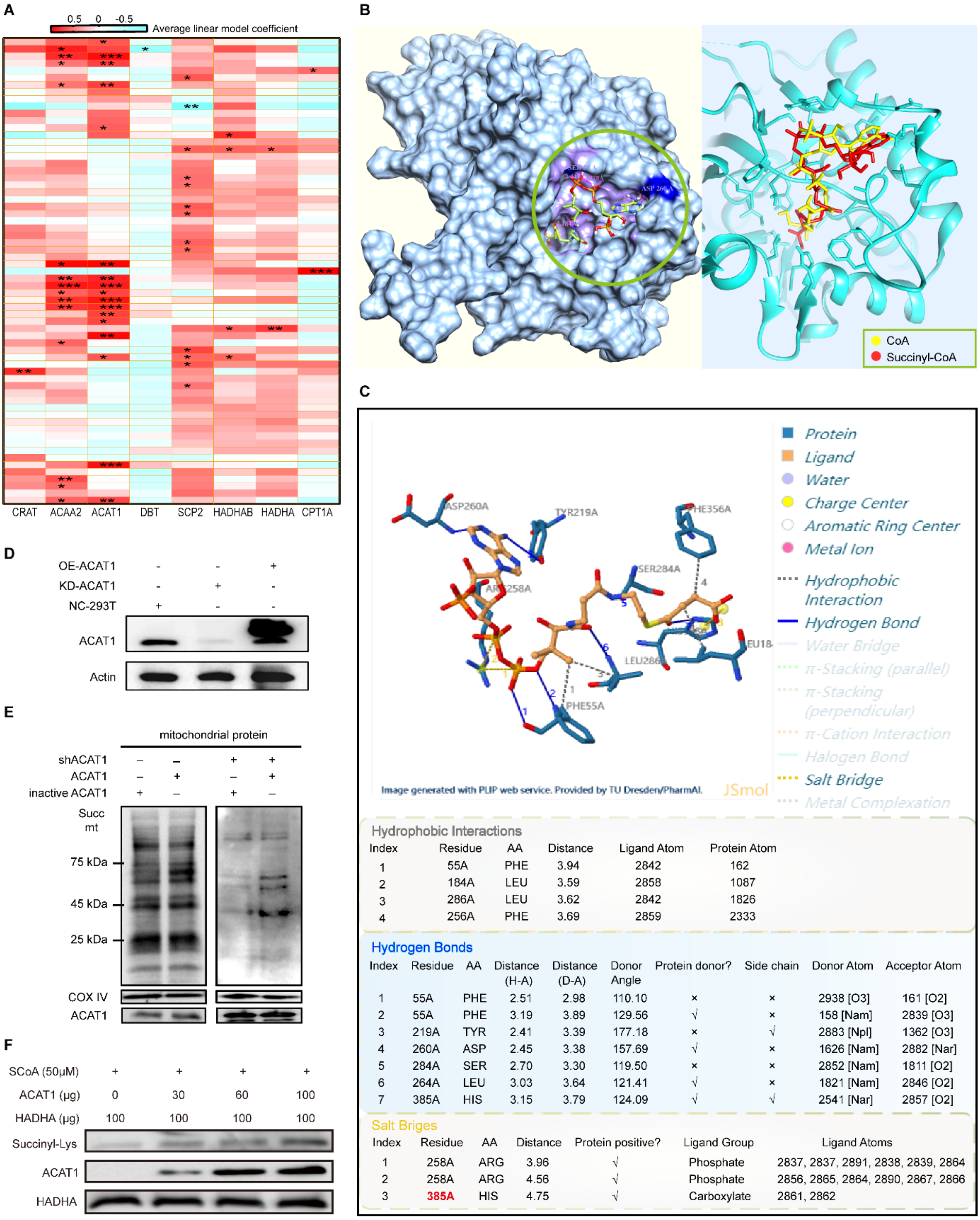

Figure S5. ACAT1 mediates mitochondrial protein hypersuccinylation. (A) Correlation analysis between the expression level of potential succinyltransferases and succinylation level of succinylation sites in the patient lung proteome and succinylation-modified proteome data. **(B)** Interactions between succinyl-CoA (SCoA) and chain A of ACAT1. SCoA is presented as rainbow-colored. Superimposition of the ACAT1-CoA and ACAT1- SCoA complex structures are shown in the right image. Models show ACAT1 in blue, SCoA in red sticks, and CoA in yellow sticks. **(C)** Details of the interactions between ACAT1 and SCoA analyzed using PLIP39. **(D)** ACAT1 expression levels in stable ACAT1 knockdown (KD) and overexpression (OE) 293T cell lines examined by IB (with HEK293T cells as a control, NC). **(E)** *In vitro* succinylation assay. Mitochondrial proteins were isolated from KD or NC cells and subjected to an *in vitro* succinylation assay using active or inactive purified His-ACAT1 in the presence of SCoA. Succinylation levels of mitochondrial proteins were detected by IB. **(F)** ACAT1 succinylates HADHA. ACAT1-mediated HADHA succinylation was analysed by mixing purified ACAT1, HADHA, and succinyl-CoA. The succinylation level of HADHA was in line with the amount of ACAT1 added to the reaction system.

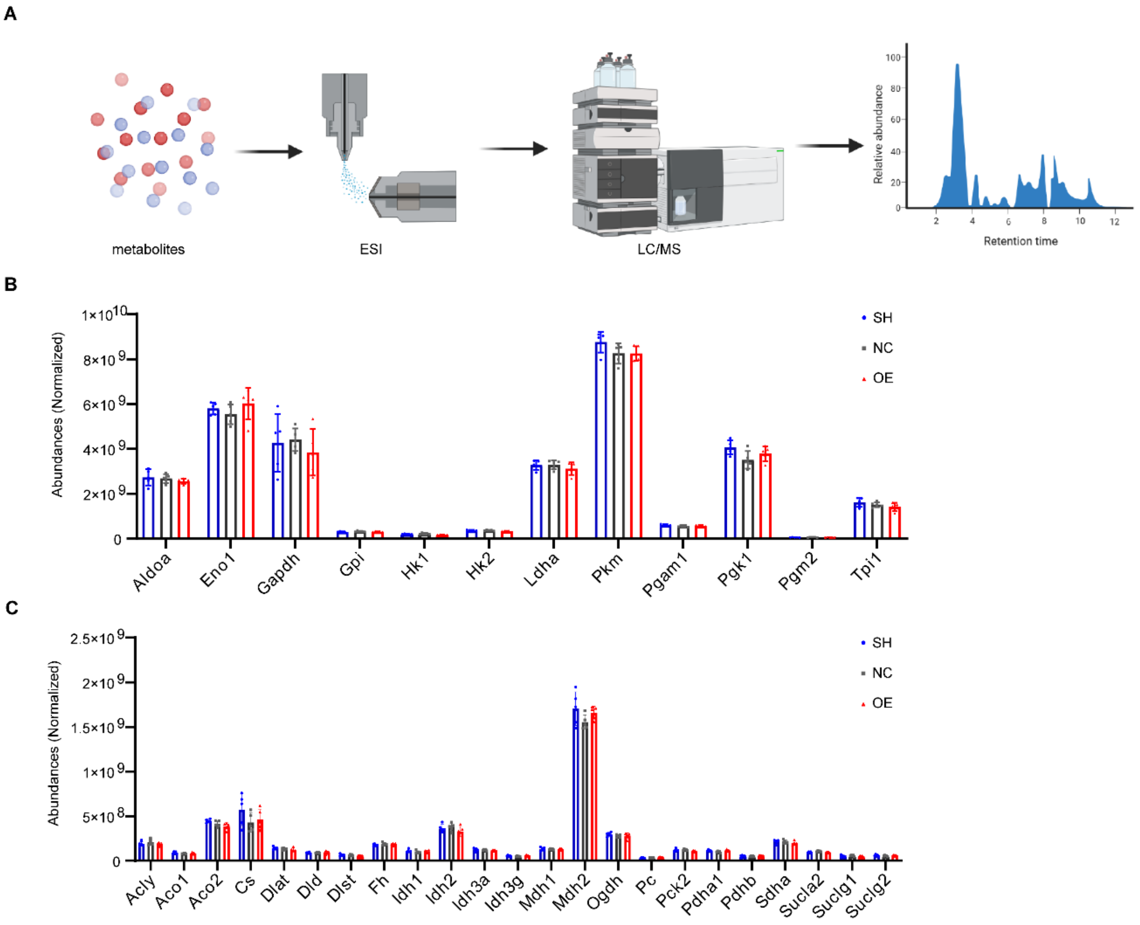

Figure S6. Correlation between ACAT1 and metabolic enzymes at the expression level. (A) Flow chart of targeted metabolism assay. **(B, C)** Expression level of proteins in glycolysis **(B)** and TCA **(C)** pathway of knockdown/negative control/overexpression Lewis lung carcinoma cell lines.

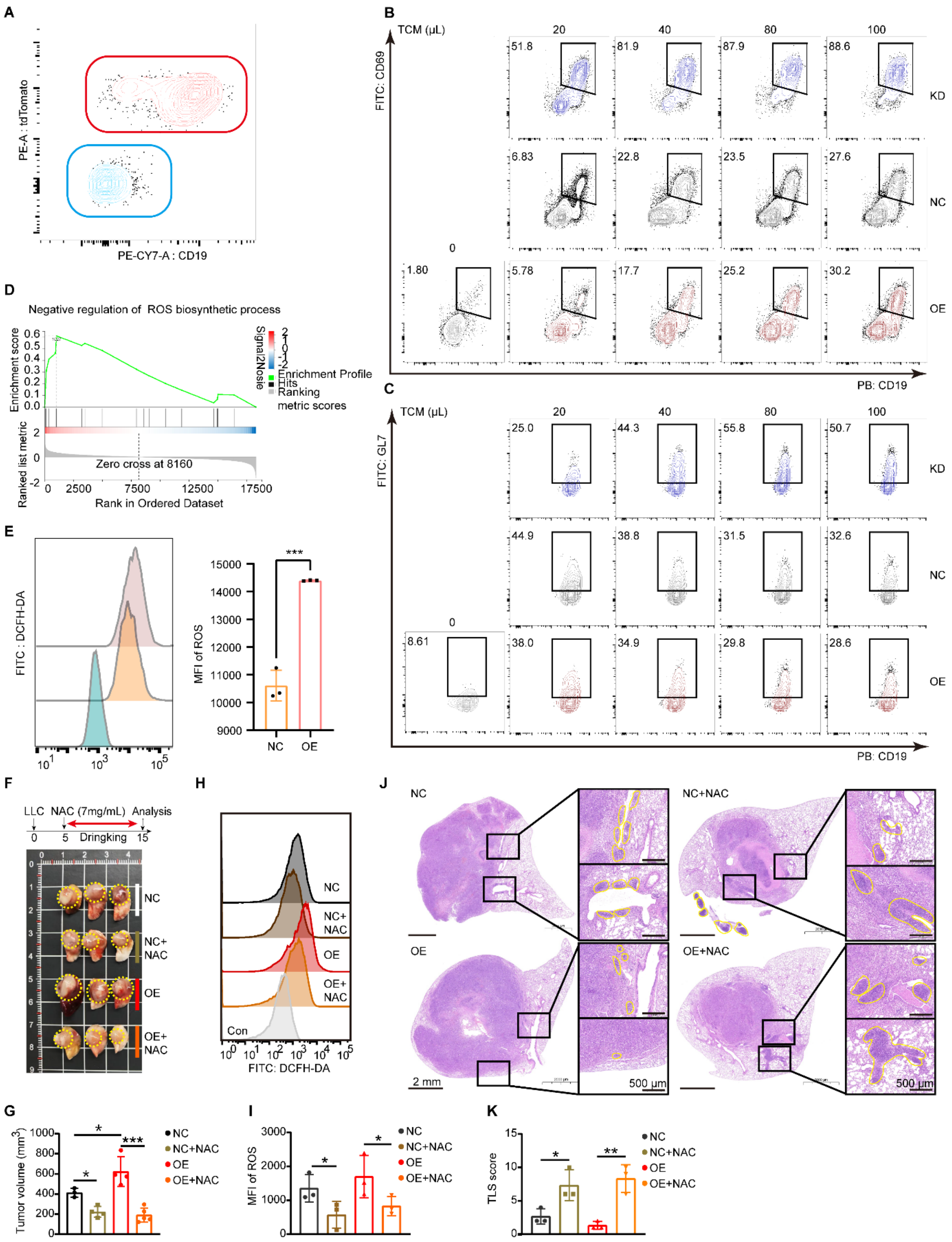

Figure S7. ACAT1 promotes ROS production of tumor cells and impedes B cell activation in the tumor microenvironment. (A) Representative scatter plots of B cells in the PBMC of Cd79aCreAi14+/- mice. **(B, C)** Representative scatter plots of Figure 7, E **(B)** and F **(C)**. **(D)** Negative regulation of ROS biosynthetic process was enriched in *Acat1* knockdown (KD) Lewis lung carcinoma cells (LLC), as determined by gene set enrichment analysis. **(E)** ROS levels of negative control (NC)/overexpression (OE) LLC cell lines, detected by 2',7'-Dichlorofluorescin Diace using flow cytometry. **(F-I)** NAC administered in drinking water was used to eliminate ROS in the orthotopic lung tumor mice models, followed by determination of tumor volume **(F-G)** and ROS clear effect **(H-I)**, $n = 4$ per group. **(J-K)** H&E staining shows the tumor site and adjacent normal tissues. TLS were marked with irregular curves in orange **(J)**. TLS score **(K)**. Data are representative of at least 2 independent experiments. Data are shown as the mean ± SD. *, P < 0.05; **, P < 0.01; ***, P < 0.001; ****, P < 0.0001. ns, not significant, with 2-tailed unpaired Student's t tests.

References

1. Trott O, and Olson AJ. AutoDock Vina: improving the speed and accuracy of docking with a new scoring function, efficient optimization, and multithreading. *J Comput Chem.* 2010;31(2):455-61.
2. Adasme MF, et al. PLIP 2021: expanding the scope of the protein-ligand interaction profiler to DNA and RNA. *Nucleic Acids Res.* 2021;49(W1):W530-W4.
3. Chen S, et al. fastp: an ultra-fast all-in-one FASTQ preprocessor. *Bioinformatics.* 2018;34(17):i884-i90.
4. Smith T, Heger A, and Sudbery I. UMI-tools: modeling sequencing errors in Unique Molecular Identifiers to improve quantification accuracy. *Genome Res.* 2017;27(3):491-9.
5. Dobin A, et al. STAR: ultrafast universal RNA-seq aligner. *Bioinformatics.* 2013;29(1):15-21.
