# Supplementary material for: ACAT1 regulates tertiary lymphoid structures and correlates with immunotherapy response in non–small cell lung cancer
Source: J Clin Invest. 2025 Apr 1;135(7):e181517. doi: 10.1172/JCI181517 (PMC11957694; doi:10.1172/JCI181517)
Supplement: Unedited blot and gel images [file jci-135-181517-s154.pdf]

Figure 5B

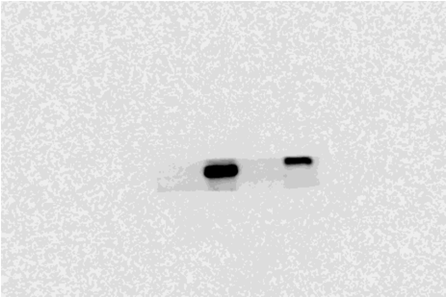

Full unedied blot for Figure5B-ACAT1

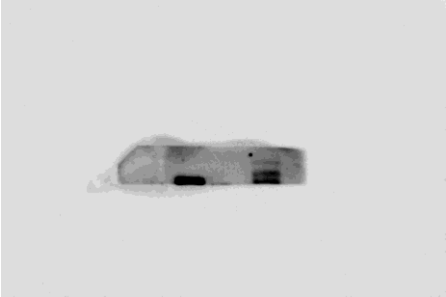

Full unedied blot for Figure5B-CS

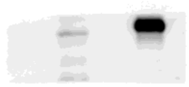

Full unedied blot for Figure5B-DLST

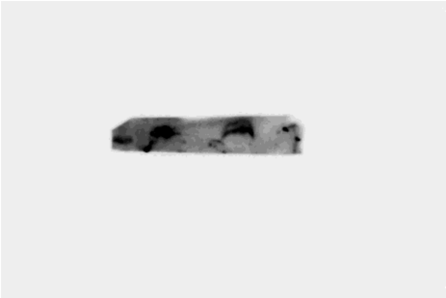

Full unedied blot for Figure5B-ETFA

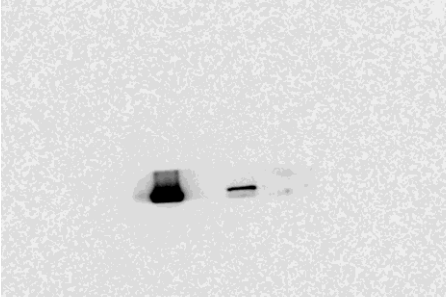

Full unedied blot for Figure5B-HADHA

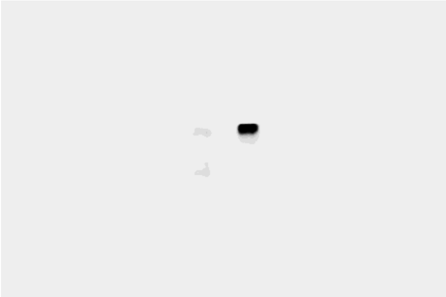

Full unedied blot for Figure5B-IDH2

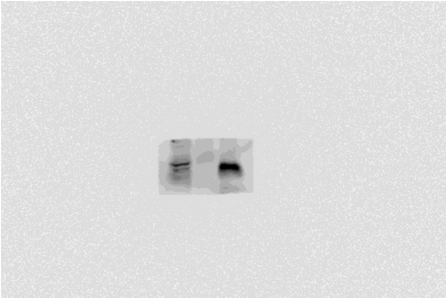

Full unedied blot for Figure5B-SHMT2

Figure 5H

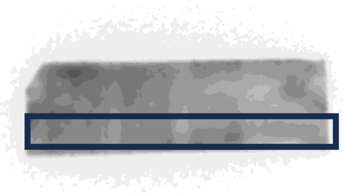

Full unedied blot
for Figure5H-Succ

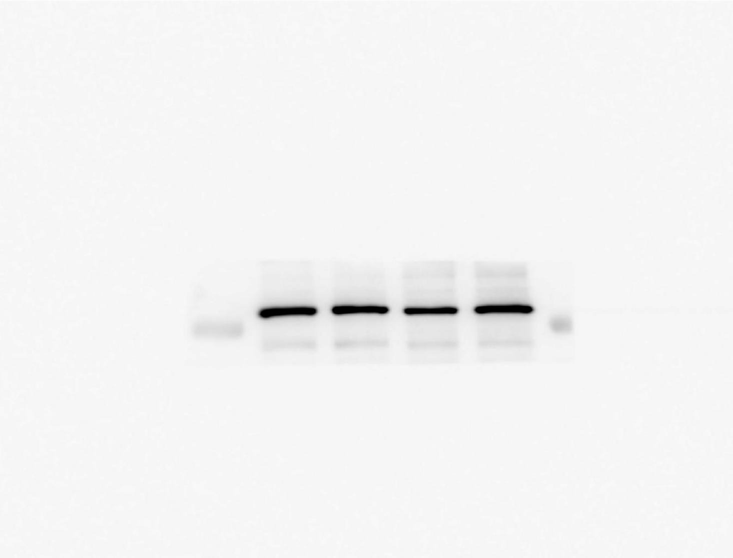

Full unedied blot
for Figure5H-HADHA

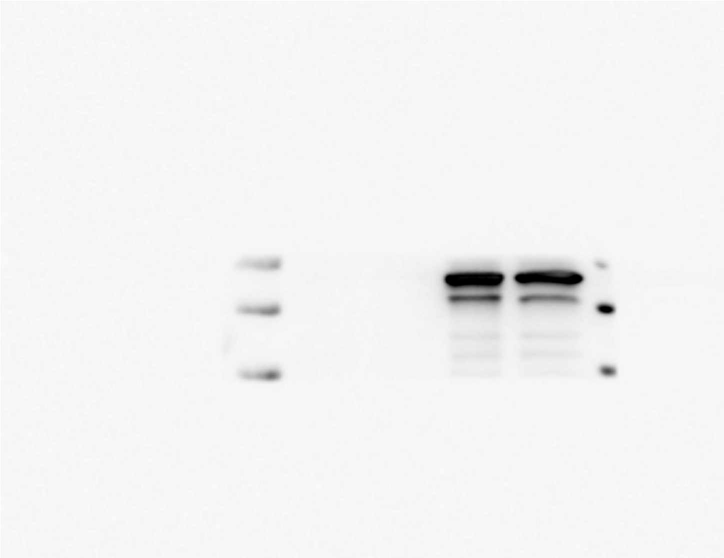

Full unedied blot
for Figure5H-ACAT1

Figure S2A

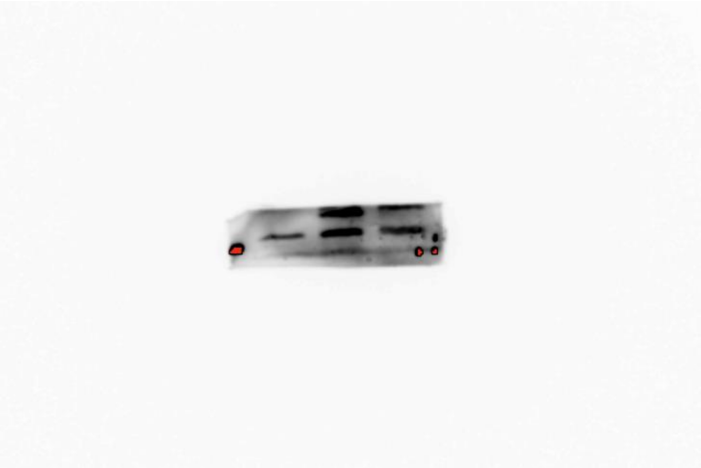

Full unedied blot for
FigureS2A-ACAT1

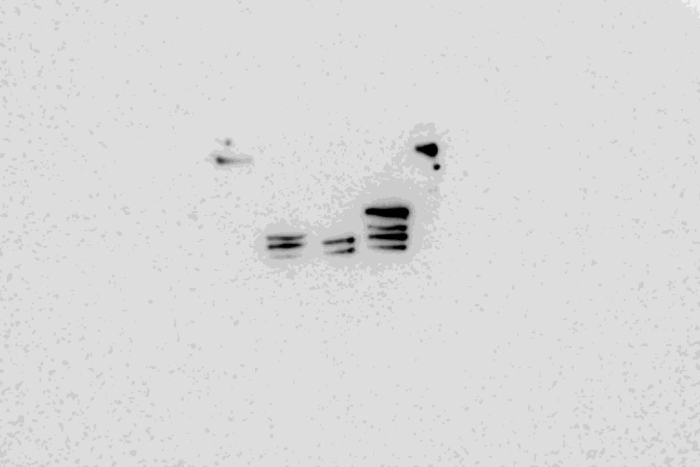

Full unedied blot for
FigureS2A-ACAT1

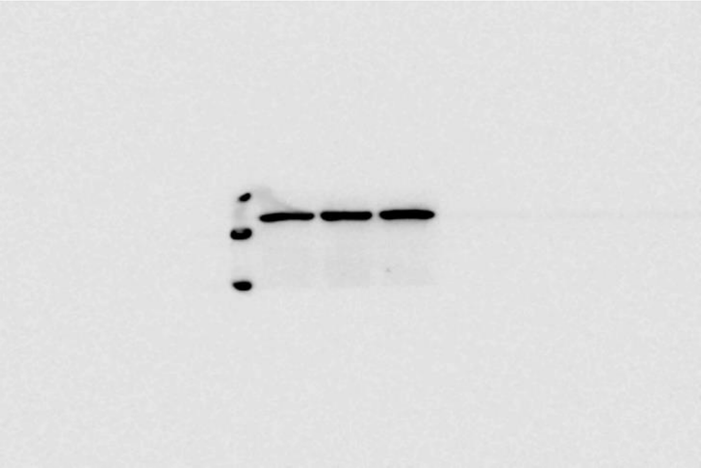

Full unedied blot for
FigureS2A-ACTIN

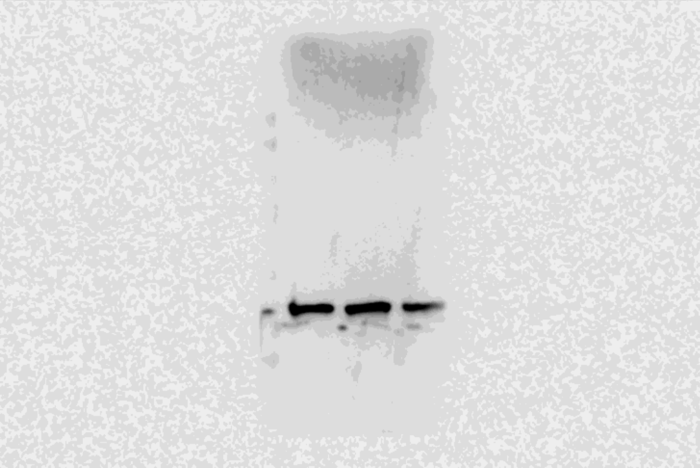

Full unedied blot for
FigureS2A-ACTIN

Figure S4C

Full unedied blot for FigureS4C-succ(mt)

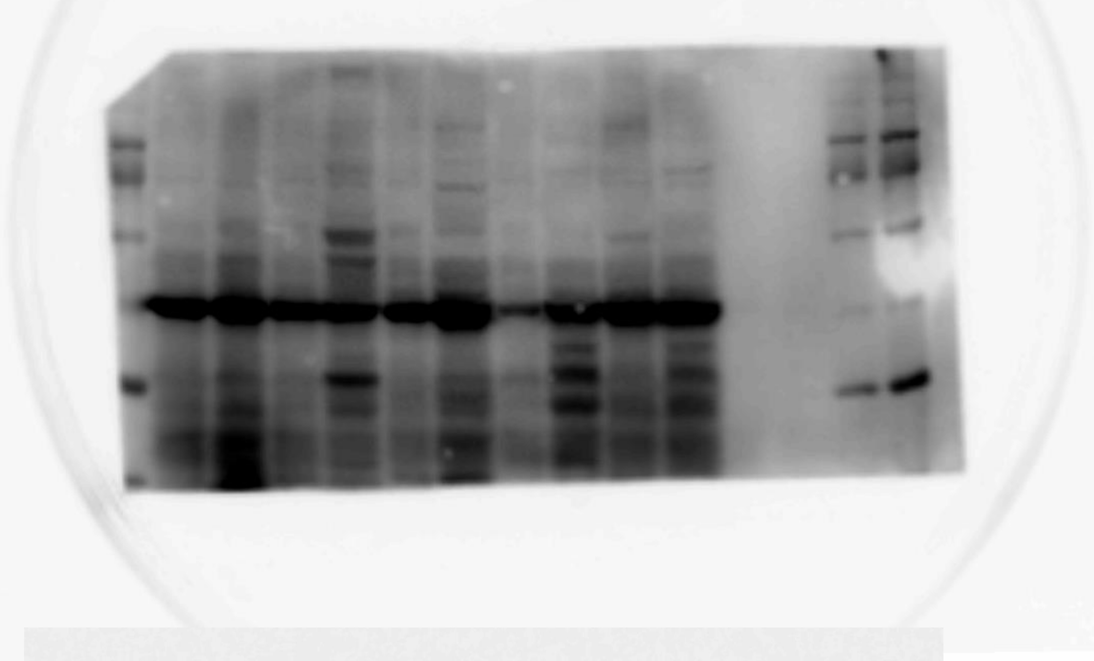

Full unedied blot for FigureS4C-ACAT1

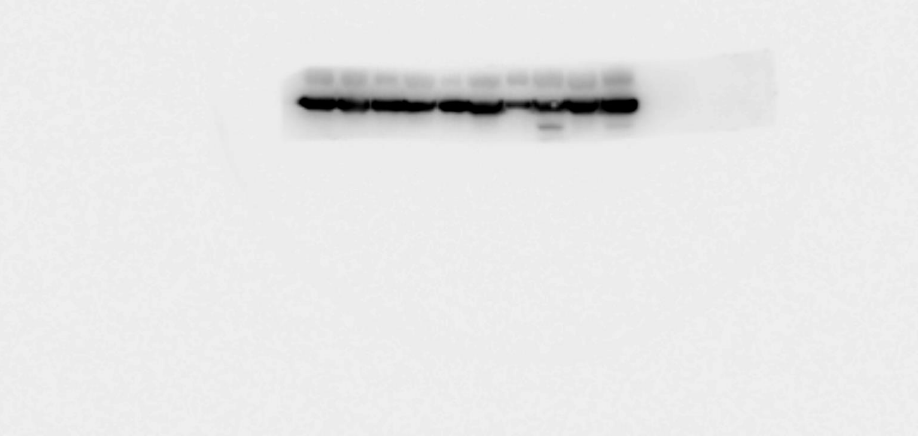

Full unedied blot for FigureS4C-COX IV

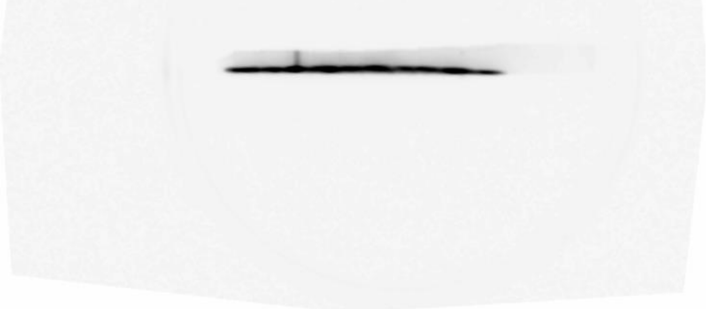

Full unedied blot for FigureS4C-HAT1

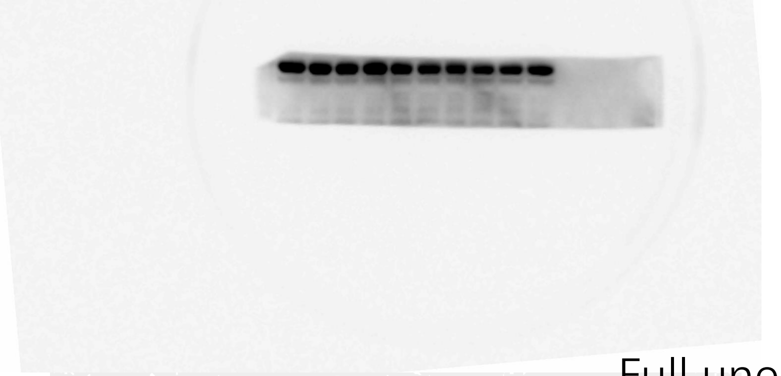

Full unedied blot for FigureS4C-CPT1A

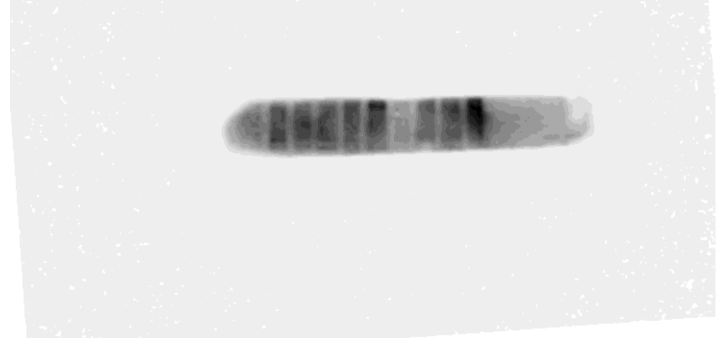

Figure S5B

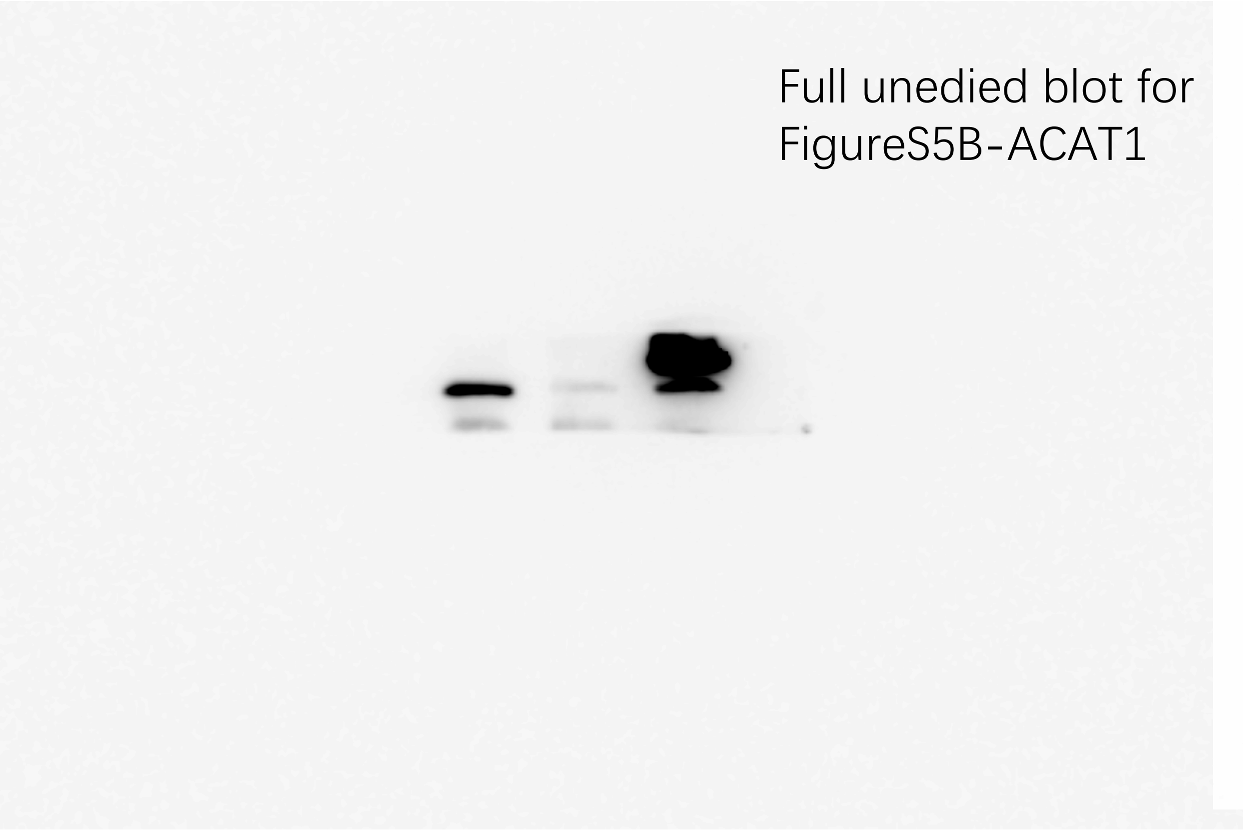
Full unedied blot for
FigureS5B-ACAT1

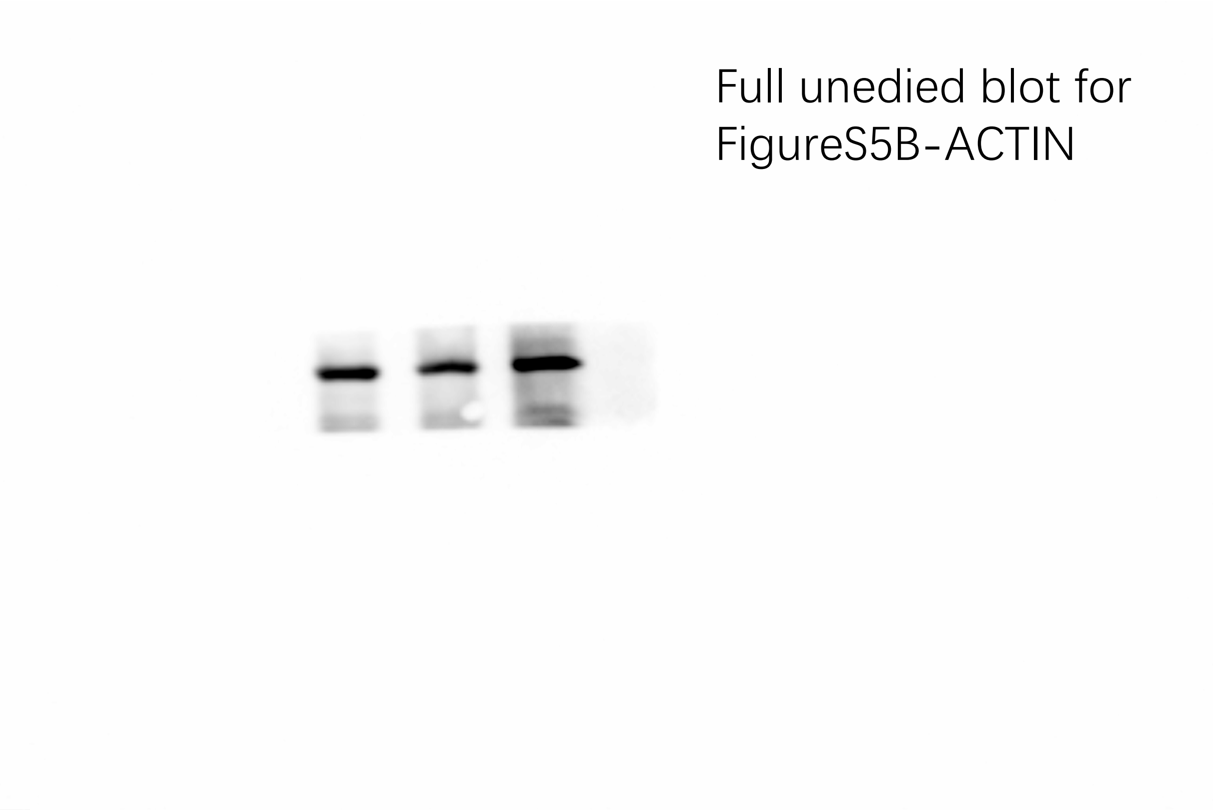
Full unedied blot for
FigureS5B-ACTIN

Figure S5E

Full unedied blot for FigureS5E-Succ

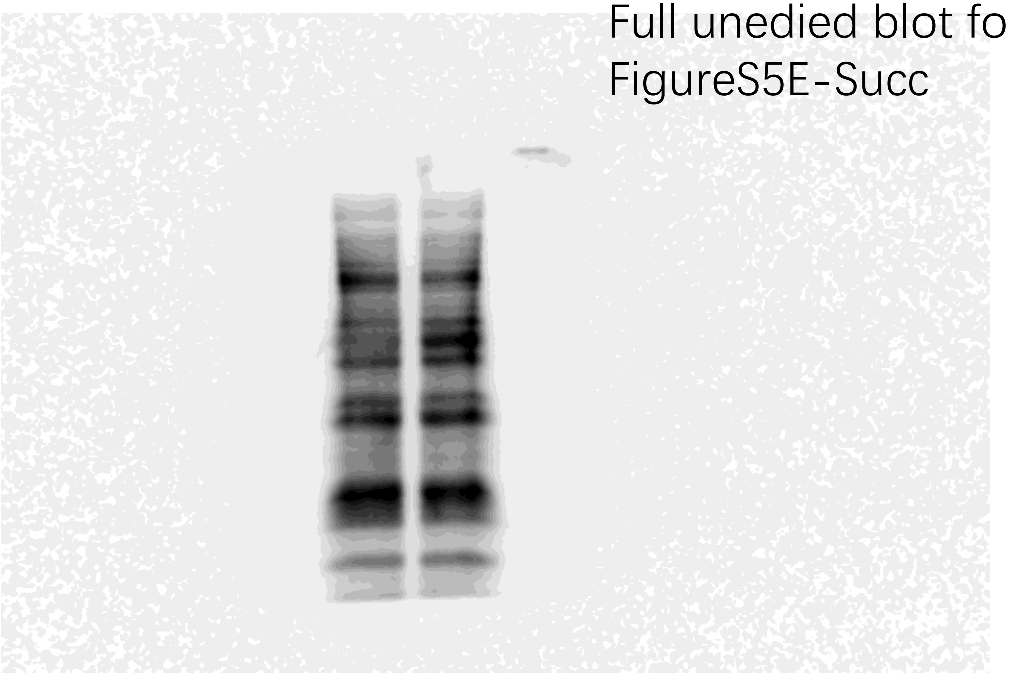

Full unedied blot for FigureS5E-Succ

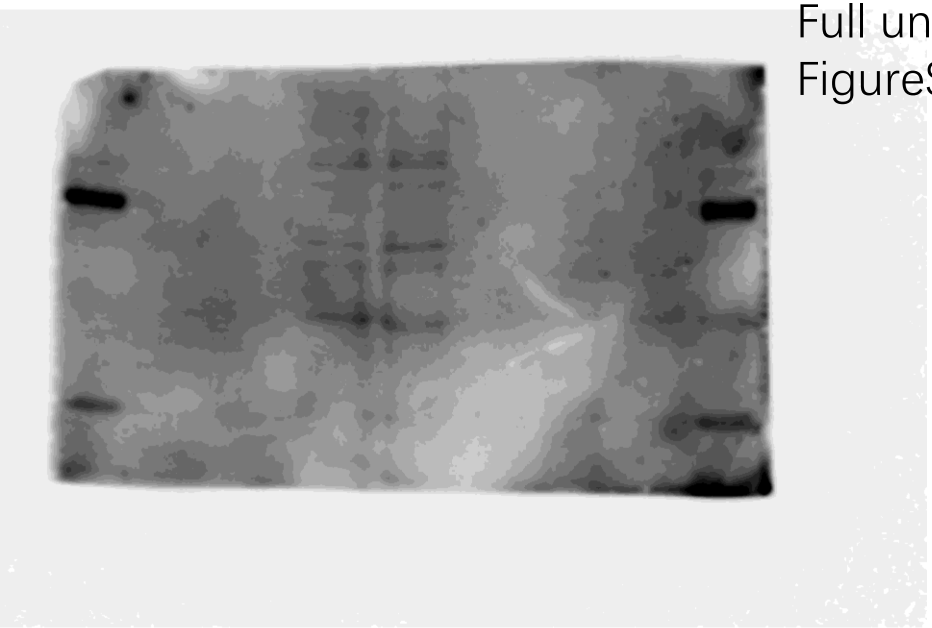

Full unedied blot for FigureS5E-COX IV

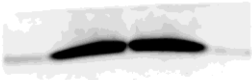

Full unedied blot for FigureS5E-ACAT1

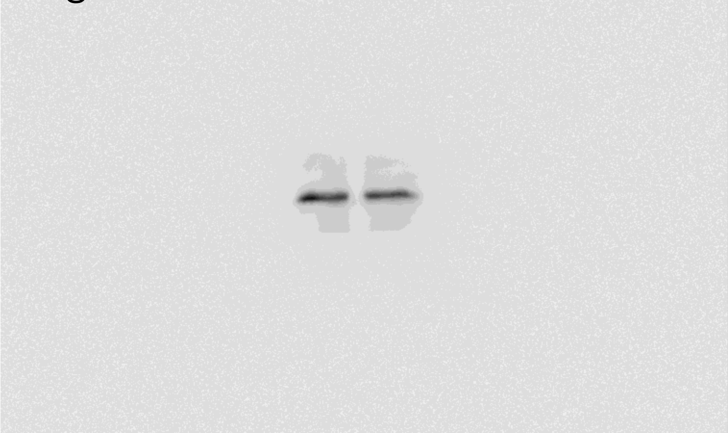

Full unedied blot for FigureS5E-COX4

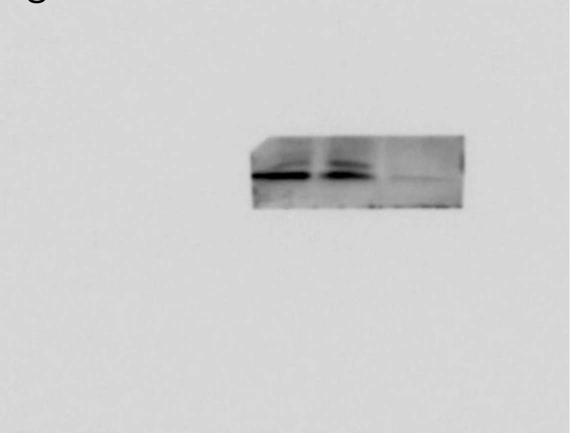

Full unedied blot for FigureS5E-ACAT1

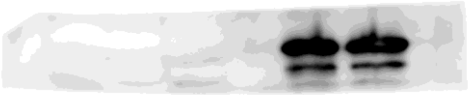

Figure S5F

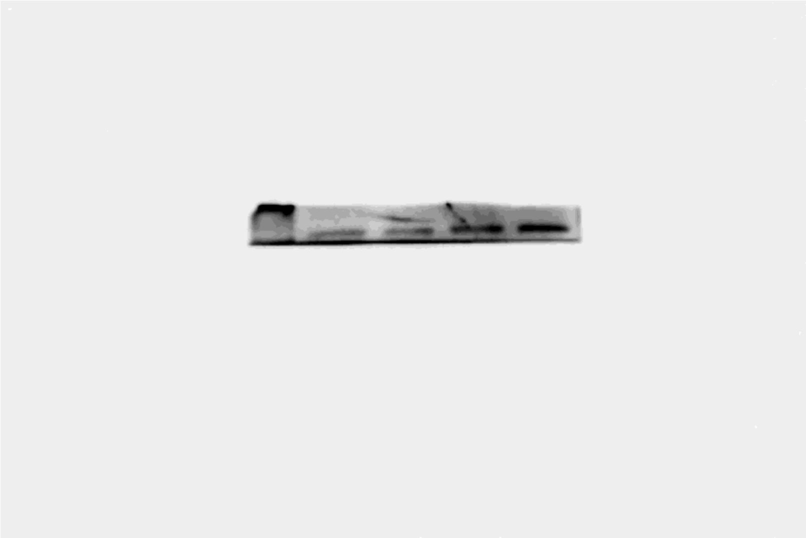

Full unedied blot
for Figure S5F-Succ

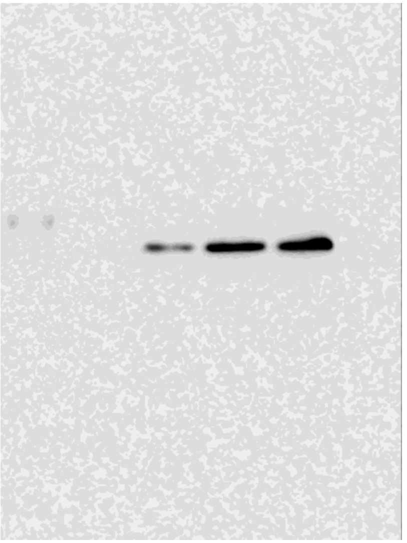

Full unedied blot
for Figure S5F-ACAT1

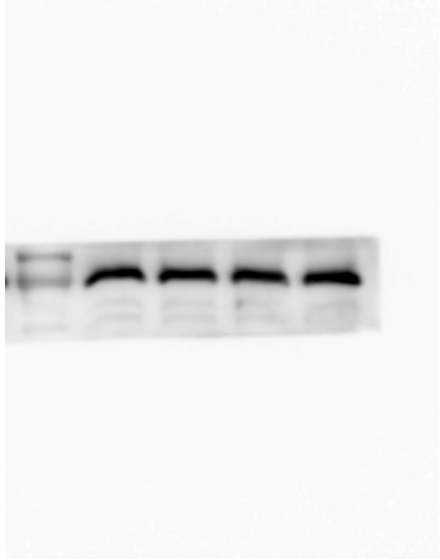

Full unedied blot
for FigureS5F-HADHA
